# Multicomponent Support Program for Secondary Prevention of Stroke Using Digital Health Technology: Co-Design Study With People Living With Stroke or Transient Ischemic Attack

**DOI:** 10.2196/54604

**Published:** 2024-08-22

**Authors:** David Silvera-Tawil, Jan Cameron, Jane Li, Marlien Varnfield, Liam P Allan, Mitch Harris, Natasha A Lannin, Christian Redd, Dominique A Cadilhac

**Affiliations:** 1 Australian e-Health Research Centre Commonwealth Scientific and Industrial Research Organisation Sydney Australia; 2 Department of Medicine Monash University Melbourne Australia; 3 Australian Centre for Heart Health Melbourne Australia; 4 Data 61 Commonwealth Scientific and Industrial Research Organisation Melbourne Australia; 5 Department of Neuroscience Monash University Melbourne Australia; 6 Florey Institute of Neuroscience and Mental Health Melbourne Australia

**Keywords:** mobile app, stroke, transient ischemic attack, health service delivery, mobile health, mHealth, mobile phone

## Abstract

**Background:**

Few individuals (<2%) who experience a stroke or transient ischemic attack (TIA) participate in secondary prevention lifestyle programs. Novel approaches that leverage digital health technology may provide a viable alternative to traditional interventions that support secondary prevention in people living with stroke or TIA. To be successful, these strategies should focus on user needs and preferences and be acceptable to clinicians and people living with stroke or TIA.

**Objective:**

This study aims to co-design, with people with lived experience of stroke or TIA (referred to as consumers) and clinicians, a multicomponent digital technology support program for secondary prevention of stroke.

**Methods:**

A consumer user needs survey (108 items) was distributed through the Australian Stroke Clinical Registry and the Stroke Association of Victoria. An invitation to a user needs survey (135 items) for clinicians was circulated via web-based professional forums and national organizations (eg, the Stroke Telehealth Community of Practice Microsoft Teams Channel) and the authors’ research networks using Twitter (subsequently rebranded X, X Corp) and LinkedIn (LinkedIn Corp). Following the surveys, 2 rounds of user experience workshops (design and usability testing workshops) were completed with representatives from each end user group (consumers and clinicians). Feedback gathered after each round informed the final design of the digital health program.

**Results:**

Overall, 112 consumers (male individuals: n=63, 56.3%) and 54 clinicians (female individuals: n=43, 80%) responded to the survey; all items were completed by 75.8% (n=85) of consumers and 78% (n=42) of clinicians. Most clinicians (46/49, 94%) indicated the importance of monitoring health and lifestyle measures more frequently than current practice, particularly physical activity, weight, and sleep. Most consumers (87/96, 90%) and clinicians (41/49, 84%) agreed that providing alerts about potential deterioration in an individual’s condition were important functions for a digital program. Intention to use a digital program for stroke prevention and discussing the data collected during face-to-face consultations was high (consumers: 79/99, 80%; clinicians 36/42, 86%). In addition, 7 consumers (male individuals: n=5, 71%) and 9 clinicians (female individuals: n=6, 67%) took part in the user experience workshops. Participants endorsed using a digital health program to help consumers manage stroke or TIA and discussed preferred functions and health measures in a digital solution for secondary prevention of stroke. They also noted the need for a mobile app that is easy to use. Clinician feedback highlighted the need for a customizable clinician portal that captures individual consumer goals.

**Conclusions:**

Following an iterative co-design process, supported by evidence from user needs surveys and user experience workshops, a consumer-facing app that integrates wearable activity trackers and a clinician web portal were designed and developed to support secondary prevention of stroke. Feasibility testing is currently in progress to assess acceptability and use.

## Introduction

### Background

Stroke is the third leading global cause of death and disability, with approximately 12.2 million incident strokes reported in 2019 [1,2]. Recurrent stroke events are frequently reported as 4% within 90 days of the index event [3] and 26% within 5 years [4]. The risk of stroke following a transient ischemic attack (TIA) is also substantial, with a 20% risk 90 days following the index event [5]. Therefore, for people who have experienced a stroke or TIA, prevention of another vascular event (ie, secondary prevention) is a priority [6,7]. Clinical guidelines recommend control of modifiable risk factors via lifestyle behavior changes in conjunction with pharmacotherapeutic measures to reduce the risk of recurrence [8,9]. These include reducing blood pressure, blood cholesterol, blood glucose, and body weight; improving diet and physical activity; and reducing or ceasing alcohol and cigarette consumption [8-10].

Interventions promoting uptake of secondary prevention strategies have been used effectively for controlling blood pressure [11] and improving diet [12], physical activity [13], and medication adherence [14]. Implementation of secondary prevention strategies largely rests in the hands of individuals who must take an active role in self-managing their health. However, health care and patient-level barriers impede uptake and adherence to these strategies. Significant improvements in the acute management of stroke have resulted in shorter hospital admissions of approximately 4 days [15], with little education and time provided for survivors to consider the long-term physical, emotional, and lifestyle behaviors needed for recovery and prevention of future events [16].

Compounding an individual’s motivation to address their vascular risk is the poor mental health experienced by many survivors of stroke [17], which often hinders their engagement in secondary prevention programs and motivation for undertaking lifestyle adaptations [18]. For example, 1 in 3 patients stop taking their antihypertension medication within the first year of stroke [19]. Consequently, a fundamental practice gap prevails with the adoption of recommended stroke prevention strategies into real-world settings [20]. Novel secondary prevention programs that are feasible to implement and are broad reaching are needed and could be realized using digital health.

The rising use of consumer devices makes mobile health (mHealth) strategies a potential solution to increase participation in secondary prevention of stroke by providing low-cost, readily accessible, and scalable interventions to promote engagement in health behavior changes [21,22]. mHealth interventions incorporating smartphone apps, wearable activity trackers, clinician portals, and electronic messaging have been used to improve the management of relevant chronic conditions associated with stroke. Findings from a systematic review indicated the use of smartphone apps, phone calls, and combined programs (>2 technologies such as SMS text messages combined with a smartphone app) improved management of hypertension, including blood pressure reduction and adherence to medication [23]. Similarly, smartphone apps have been linked to improved management of diabetes mellitus [24] as well as to enhanced management of chronic heart disease when used alongside SMS text messages, phone calls, and pedometers [25,26]. Their impact includes improving clinical cardiovascular outcomes (eg, blood pressure control and medication adherence) [23-26] as well as psychosocial outcomes [26]. These interventions have also been successfully implemented in the real world. Cardihab (Cardihab Pty Ltd), for example, combines a smartphone app with a clinician-facing web portal to deliver cardiac rehabilitation to patients after a myocardial infarct [27]. When evaluated in a randomized controlled trial, Cardihab improved functional walking capacity, psychosocial health, weight, and quality of life [26].

Despite the potential benefits of mHealth for risk-factor reduction and chronic disease management, evidence-based approaches for secondary prevention of stroke are still nascent, and further research is required to inform the development of platforms that are acceptable and appropriate for people living with stroke or TIA [28]. Furthermore, development of these programs must include considerations of accessibility for those with less access to technology, lower digital literacy, and ongoing impairments after stroke [28].

### Objectives

Using a co-design approach, we aimed to develop a novel digital secondary prevention program with people living with stroke or TIA (consumers) and clinicians. The aim of this program is to offer an easily accessible solution that supports secondary stroke prevention, empowers self-management, and enhances health outcomes. The CAPS (Care Assistant and Support Program for People After Stroke or TIA) program draws upon past successes of the cloud-based Mobile Technology–Enabled Rehabilitation platform [26,29] and an electronic interface for scheduling electronic health messages aligned to individual goals for recovery and secondary prevention of stroke (inspiring Virtual Enabled Resources following Vascular Events) [30].

We report here on the user needs analysis and user experience workshops. Finally, we present the resulting components of the mHealth platform (ie, mobile app and clinician portal) and care management program that uses lifestyle and health data collected through a wearable device to facilitate secondary prevention for individuals after stroke or TIA.

## Methods

The design of the CAPS program and digital platform were completed using a mixed methods approach, undertaken in 2 main stages: (1) a user needs analysis to explore the main requirements, priorities, and potential use of digital technology to support secondary prevention of stroke or TIA and (2) user experience workshops to inform the design of the main components of the digital health program.

### Stage 1: User Needs Analysis

The requirements and challenges associated with the creation of CAPS were investigated via researcher-developed surveys completed between December 2020 and April 2021. Particular attention was given to the potential use of 2 broad digital technologies: mobile apps and wearable or health monitoring devices.

#### Participants

User needs surveys were completed by people with lived experience of stroke or TIA (referred here on as consumers) and clinicians who provide health care and education services to individuals after stroke or TIA. These populations have lived experience of stroke (and care for patients after stroke) and are well placed to comment on the needs, preferences, and perceived potential of secondary prevention programs.

Consumer participants were invited to participate via invitation letters posted to 840 registrants of the Australian Stroke Clinical Registry (AuSCR), a national clinical quality registry of patients admitted to participating hospitals with a diagnosis of acute stroke or TIA [31]. The AuSCR consists of >35,000 Australian survivors of stroke who are known to be alive and willing to be contacted to participate in research projects. Once the proposal was reviewed, the AuSCR staff distributed invitations to registrants based on the study eligibility criteria. Invitation letters were also distributed through the Stroke Association (Victoria, Australia), which operates 5 support centers across regional Victoria, offering dedicated community-based support services for people recovering from stroke. These methods were used to improve response rates by directly inviting potentially eligible participants from a cohort that has previously been hard-to-reach [32,33].

Eligibility criteria included a diagnosis of stroke or TIA in the past 6 months to 5 years; aged ≥18 years; proficiency in spoken and written English; and, for AuSCR participants, those who agreed to be contacted by the AuSCR for future research. Individuals residing in a nursing home or those with no access to the internet were ineligible to participate.

Invitations to clinicians were circulated via the authors’ research networks, professional forums, and national organizations. We sought responses from an unbiased and representative sample, recruiting through Twitter (subsequently rebranded X, X Corp) and LinkedIn (LinkedIn Corp) on personal research accounts (>1200 followers, many of whom shared the original tweet) and the Stroke Society of Australia (>7000 members), who also shared the invitation via Twitter and email. We also used the Australian Stroke Telehealth Community of Practice Microsoft Teams Channel, with >500 clinicians and researchers that use the group as a forum to exchange ideas and knowledge. Participation in the surveys was anonymous.

Eligibility included all clinicians who provide health care and education services to individuals after stroke or TIA. Participant eligibility was confirmed via the demographics questionnaire in each survey, including self-reported clinical history (for consumers) or self-reported clinician expertise.

#### Survey Development

Consumer experience and perceptions were explored in three dimensions: (1) the participants’ experience and interests in mobile apps and wearable and health monitoring devices, (2) their preferred functions and priorities around health measures required in a digital support program for secondary prevention of stroke, and (3) their attitude toward a digital support program for secondary prevention of stroke. The consumer survey consisted of 28 questions, with a total of 108 items (Multimedia Appendix 1).

Similarly, clinicians’ experience and perception were explored in three dimensions: (1) existing health measures commonly collected in the management of people with stroke and TIA, (2) the participants’ priorities around health measurements required in a digital support program for secondary prevention of stroke, and (3) their attitude toward a digital support program for secondary prevention of stroke. The clinician survey comprised 16 questions, with a total of 135 items (Multimedia Appendix 2).

In both surveys, the questions related to the participants’ experience and interest in mobile apps, wearable and health devices, as well as their preferred functions for a digital support program were adapted from surveys developed and previously used in mHealth research [26,29,34]. The questions related to the priorities around health measures commonly collected and proposed for a digital support program were also informed by previous mHealth research [26] and clinical guidelines for secondary prevention [8,9]. Finally, the constructs of attitude toward a digital support program were adapted from the technology acceptance model, previously validated and commonly used in mHealth studies with both patients and clinicians [35-37].

While the constructs of the consumer and clinician surveys are similar, differences exist due to the roles and experience of each participant group. Therefore, we aimed to focus more attention toward the consumer preferences and engagement with a digital program, while clinician surveys focused on clinical value (eg, relevant data collection) and usability. The final surveys were tested for clarity by 4 adults (2 of them with clinical experience) before they were shared with participants.

#### Data Collection

Clinician surveys were collected using a web-based survey. For consumers, a study pack was posted to potential participants, including a participant explanatory statement and consent form, a paper-based survey with a return prepaid envelope, and a link to a web-based survey. Consumer participants were asked to complete only 1 version of the survey, either web- or paper-based, depending on their preference.

The web-based survey was captured using the REDCap (Research Electronic Data Capture; Vanderbilt University), a secure software platform designed to support data capture for research studies [38,39].

### Stage 2: User Experience Workshops

#### Overview

The aim of this stage was to complete the co-design of the CAPS program and digital platform. Workshops were conducted via teleconference (Webex, Cisco Systems Inc) in 2 rounds. The first round (design workshops), completed between April and May 2021, focused on confirming insights from the user needs surveys and gathering design requirements from participants to develop initial prototypes of CAPS, while the second round (usability testing), completed between October and November 2021, focused on collecting usability feedback on the prototypes to inform the final design.

#### Participants

Purposive sampling was used in recruiting participants. For the design workshops, all survey participants who indicated their willingness for participation in future research were considered. The final selection process was designed to achieve a balanced sample based on participants’ age, sex, diagnosis (for consumer participants), and professional roles (for clinician participants). In addition, an invitation was sent to the Stroke Foundation of Australia requesting for a lived experience representative and advocate to attend a workshop. Consumer participants were further screened via a phone call to include participants with different levels of familiarity with mobile apps and those able to connect remotely (via phone or videoconference).

For the usability testing, all participants from the design workshops were invited to participate. Invitations were also extended to a selected number of survey participants following the same criteria used in the design workshops.

#### Procedure

The user experience workshops were completed in three steps: (1) results from the stage 1 surveys were used to generate discussion topics for the design workshops; (2) findings from the design workshops were used to inform the design of wireframes and a web-based clickable prototype of a user-facing app and clinician portal; and (3) the clickable prototypes were shared with participants during the usability testing workshops to give them the opportunity to explore some of its features and provide feedback.

All workshop sessions were conducted separately with consumer and clinician participants. Workshop sessions lasted approximately 30 to 60 minutes, depending on the number of participants involved in the session. Each session was facilitated by 2 researchers. All sessions were audio recorded using Webex.

#### Data Analysis

Quantitative survey data were summarized and analyzed with Excel (Microsoft Corp) and Python (version 3.0; Python Software Foundation), using descriptive statistics. Ranking questions, where respondents ranked multiple items in order of preference, were calculated using weighted averages [40-42], as shown in equation 1:



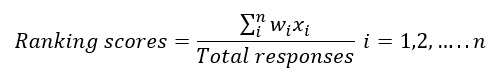



in which *w* is the ranked position for each answer choice as selected by a participant, *x* is the response count for each answer choice, and *n* is the number of choices. Each participant’s preferred choice (ranked as 1) has the largest weight *n*, and their least preferred choice has a weight of 1.

Qualitative data from the workshops were auto transcribed in Webex and cross-checked for accuracy, reviewed, and independently analyzed for themes by 2 researchers using Excel. An inductive approach was used to determine the coding of the data guided by the dimensions explored in the surveys and with a focus on design needs and preferences. There were no significant discrepancies between the reviewers. The final themes and subthemes (eg, design needs and preferences) were critically reviewed and discussed by the research team who addressed any discordance in the coding through consensus.

To avoid a misleading sense of statistical validity given the nonrandom purposive sample and the semistructured nature of workshops [43,44], we refrain from reporting qualitative results using whole numbers or percentages. Instead, to provide a sense of generalizability, we describe findings as emerging from all (100%), (approximately 76%-99%), many (approximately 51%-75%), some (approximately 25%-50%), or a few (<25%) participants.

### Ethical Considerations

This project was conducted with ethics approval from the Monash University (Human Research Ethics Committee, HREC 24806) and the Commonwealth Scientific and Industrial Research Organisation (HREC 2020_076_RR). All participants provided written informed consent before their participation. Participants who completed the web-based survey (both consumers and clinicians) were directed to a digital explanatory statement and consent form before the start of the survey.

Participation in the surveys was anonymous. However, after completion of the survey, participants were given the option to provide their contact details if they were willing to be further involved in the research program. This information was stored in a separate REDCap database unlinked from the survey responses. Workshop participants received an AUD $50 (US $33.82) gift card for their participation.

## Results

### Stage 1: User Needs Analysis

#### Participant Summary

A total of 112 consumers (male consumers: n=63, 56%) aged between 40 and 98 years completed the survey (Table 1). Of the 112 consumers, 16 (14.3%) responded to the web-based survey and the rest responded via post. A total of 101/112 (90.2%) answered at least 1 item in all questions and 85 (76%) completed all items. Most of them (110/112, 98.2%) were registrants of the AuSCR, and only 1.8% (2/112) of them responded to the invitation distributed through the Stroke Association of Victoria.

Most participants had lived experienced of stroke (72/112, 64.3%) within the last 5 years, were taking medications to manage their symptoms of stroke or reduce the risk of a future stroke (104/112, 92.9%), were not seeing a health professional to support their recovery or ongoing management after stroke (71/112, 63.4%), and were not participating in a stroke or TIA rehabilitation program (108/112, 96.4%).

A total of 54 individuals (female: n=43, 80%) with an average of 17 years of experience since finishing their entry-level clinical training responded to the clinician survey. Most of them (42/54, 78%) answered all questions. A further 24 participants answered only demographic questions and were not included in further analysis (Table 2).

**Table 1:** Characteristics of the consumer participants who have experienced a stroke or transient ischemic attack (n=112).

Characteristics			Values
**Demographics**			
	**Sex, n (%)**		
		Male	63 (56.3)
		Female	45 (40.2)
		No answer	4 (3.6)
	Age (y), mean (SD)		71.94 (10.25)
	**Education, n (%)**		
		≤Year 12	68 (60.7)
		Vocational qualification	11 (9.8)
		Associate diploma	2 (1.8)
		Undergraduate studies	12 (10.7)
		Postgraduate studies	15 (13.4)
		No answer	4 (3.6)
	**Living arrangements, n (%)**		
		Alone	30 (26.8)
		With others	78 (69.6)
		No answer	4 (3.6)
**Medical history**			
	Years since most resent stroke or TIA^a^, mean (SD)		2.65 (0.86)
	**Stroke or TIA, n (%)**		
		Stroke	72 (64.3)
		TIA	29 (25.9)
		Don’t know	11 (9.8)
	**Participating in a stroke rehabilitation program, n (%)**		
		Yes	4 (3.6)
		No	106 (94.6)
		No answer	2 (1.8)
	**Receiving a home rehabilitation program, n (%)**		
		Yes	2 (1.8)
		No	108 (96.4)
		No answer	2 (1.8)
	**Seeing a health professional to support stroke management, n (%)**		
		Yes	39 (34.8)
		No	71 (63.4)
		No answer	2 (1.8)
	**Take medications to manage stroke or TIA, n (%)**		
		Yes	104 (92.9)
		No	6 (5.4)
		No answer	2 (1.8)

^a^TIA: transient ischemic attack.

**Table 2:** Demographics and characteristics of the clinician participants. A total of 78 participants answered all questions, with 24 who answered only demographic questions being excluded from further analysis.

Demographics and characteristics		Participants (n=54)	Removed participants^a^ (n=24)
**Sex, n (%)**			
	Male	11 (20)	5 (21)
	Female	43 (80)	19 (79)
**Age (y), n (%)**			
	≤29	7 (13)	1 (4)
	30-39	18 (33)	9 (38)
	40-49	15 (28)	4 (17)
	50-59	11 (20)	9 (38)
	60-69	3 (6)	0 (0)
	70-79	0 (0)	1 (4)
Years of experience, mean (SD)		16.76 (10.13)	17.58 (9.86)
Percentage of time providing stroke or TIA^b^ care, mean (SD)		54.77 (34.88)	58.1 (33.59)
People with stroke or TIA treated per week, mean (SD)		9.62 (8.03)	10.75 (7.52)
**Profession, n (%)**			
	Medical doctor^c^	13 (24)	4 (17)
	Occupational therapist	12 (22)	0
	Physiotherapist	8 (15)	8 (33)
	Nurse	12 (22)	6 (25)
	Speech pathologist	4 (7)	1 (4)
	Social worker	1 (2)	2 (8)
	Other^d^	4 (7)	3 (13)
**Clinical setting, n (%)**			
	Acute hospital	25 (46)	9 (38)
	Community-based service	5 (9)	5 (21)
	Primary health care	1 (2)	1 (4)
	Rehabilitation	19 (35)	4 (17)
	Other	4 (7)	5 (21)

^a^Participants who only completed the demographic questions and were removed from further analysis.

^b^TIA: transient ischemic attack.

^c^Includes neurology, cardiology, rehabilitation, and general medicine.

^d^Includes stroke coordinator, radiographer, and exercise physiologist.

#### Experience Using Mobile Apps

**Figure 1:**
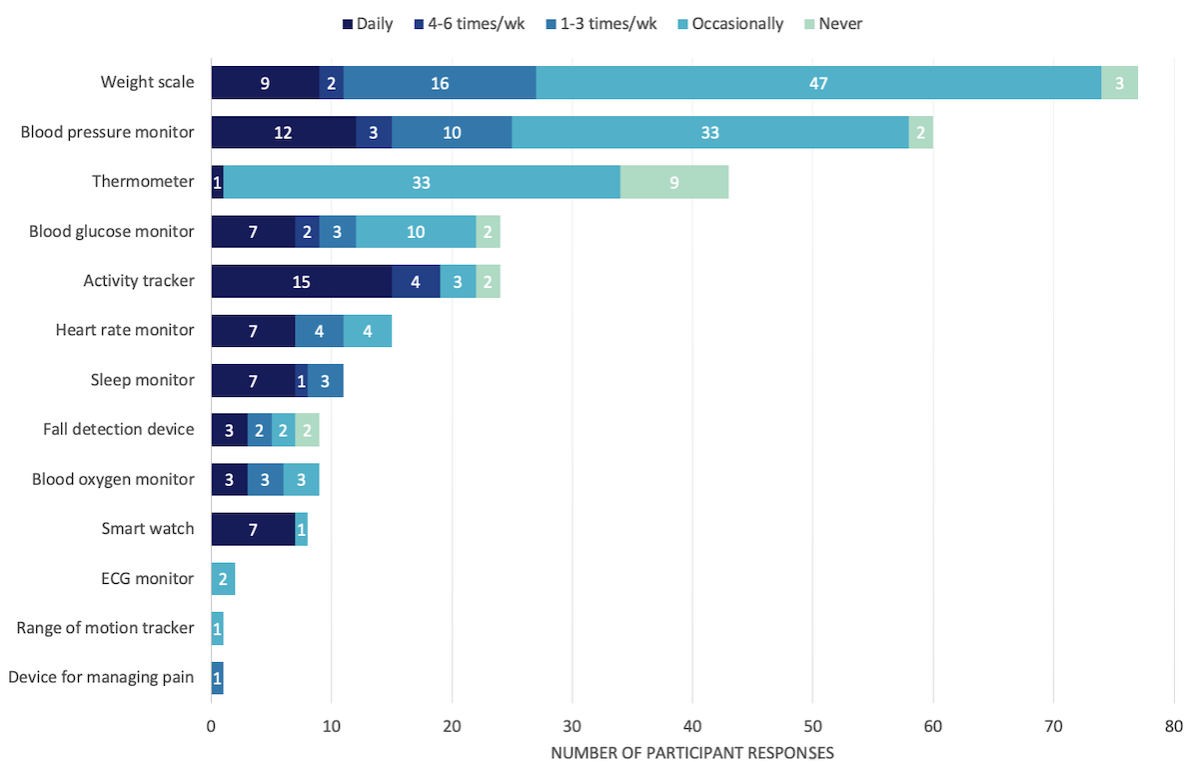
Total of the current device use by consumer participants (N=112). ECG: electrocardiogram.

A total of 81.2% (91/112) of the consumers reported using smartphones or tablets, with 58 (64%) of them using apps on a regular basis and 22 (38%) of those using apps to manage their health and well-being, particularly to track their exercise and activity levels (n=20, 35%). Many of them reported owning and using a weight scale (74/112, 66.1%) and blood pressure monitor (58/112, 51.8%), and only 26.8% (30/112) reported owning and using a smart watch or fitness tracker (Figure 1). From a clinician perspective, only 37% (20/54) of the clinicians have recommended their patients mobile apps to manage their health and well-being, particularly for physical activity monitoring (14/20, 70%) and mental health support (8/20, 40%).

#### Preferred Functions

**Figure 2:**
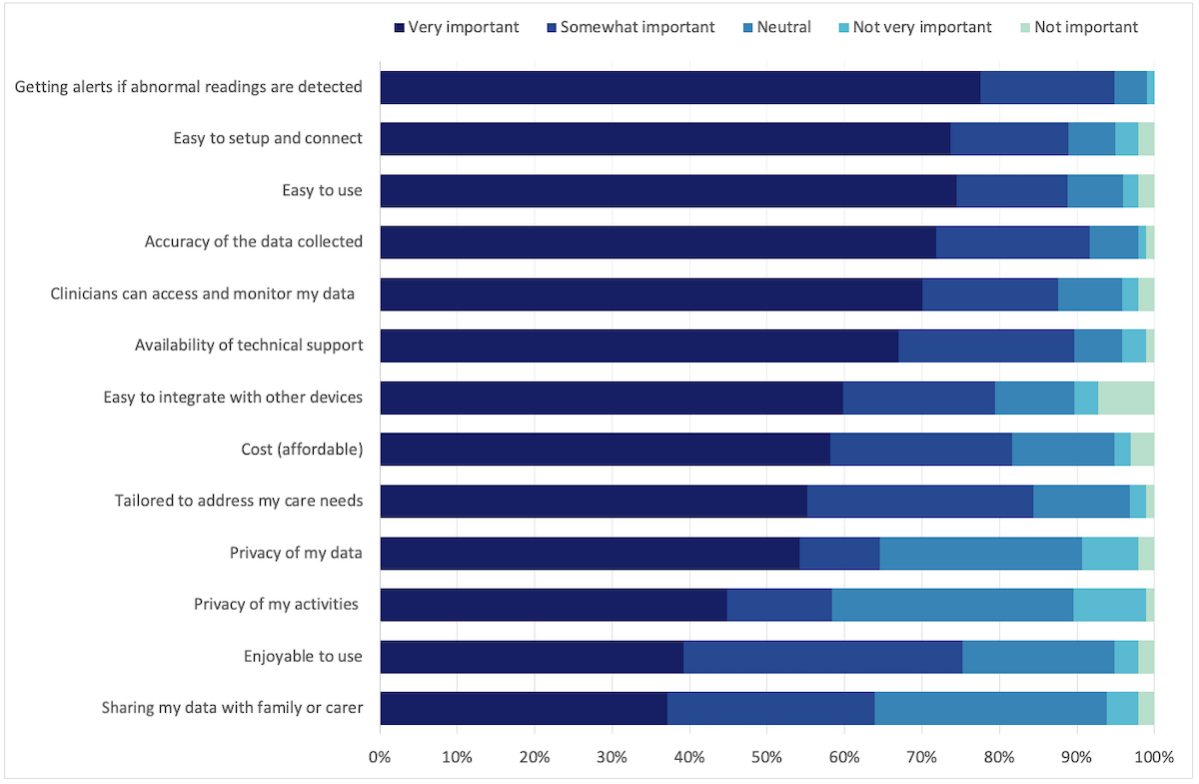
Consumer participants’ ratings on the main considerations for a digital support program for people after stroke or transient ischemic attack (N=112).

**Figure 3:**
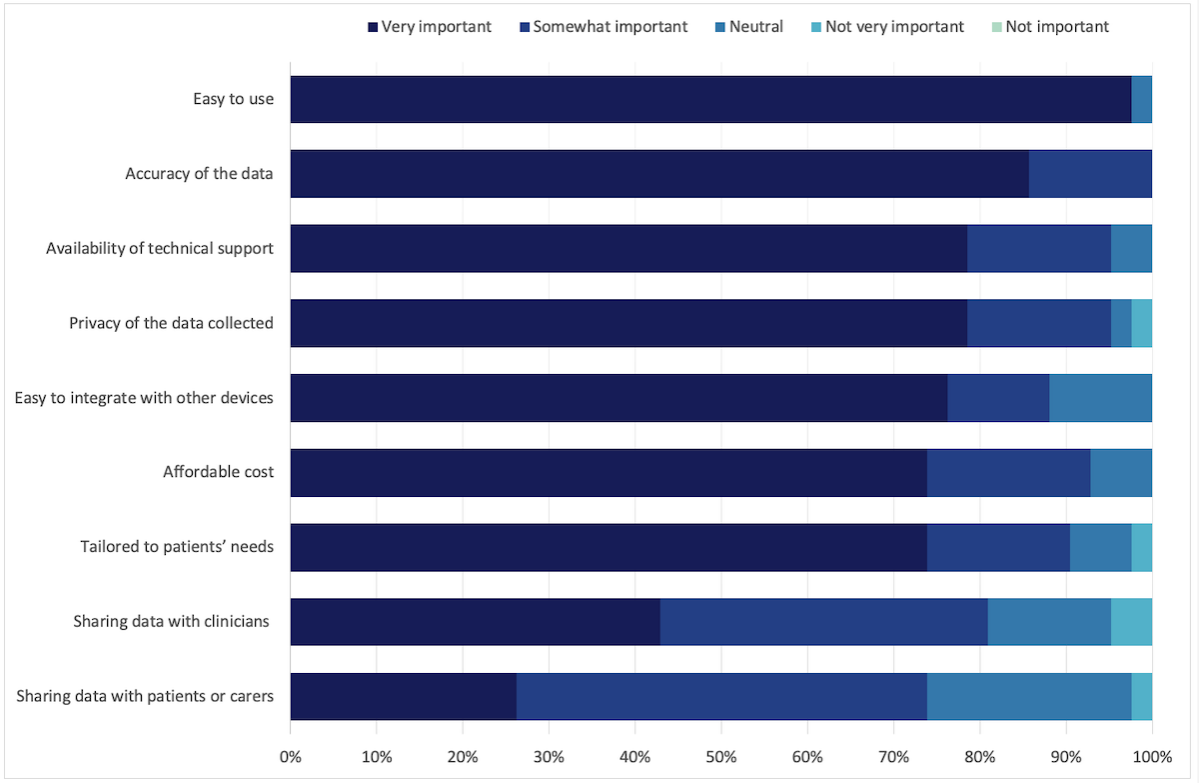
Clinician participants’ ratings on the main considerations for a digital support program for people after stroke or transient ischemic attack (n=42).

When asked about their preferred functions to include in a digital support program for stroke or TIA, consumers and clinicians prioritized two main functions (Table 3): (1) providing alerts to patients about potential deterioration in their condition and (2) collecting and monitoring lifestyle measures (eg, steps, sleep, and exercise). They also prioritized data accuracy and the need for a program that was easy to set up and use (Figures 2 and 3).

**Table 3:** Top 15 functions to be included in a digital support program for people after stroke or TIA^a^, as ranked by participants. Consumers ranked function from 1 (most important) to 3, while clinicians ranked them from 1 (most important) to 5.

Preferred function	Consumer rankings	Clinician rankings
Alerting patients about potential deterioration in their condition	1.72	2.04
Collection and monitoring lifestyle measure (eg, steps and sleep)	0.52	1.76
Tools to manage new lifestyle (eg, exercises, diet, alcohol, and smoke)	0.17	1.67
Educational information in general about stroke or TIA treatment and prevention	0.27	1.55
Alerting clinicians about potential deterioration in a patient’s condition	—^b^	1.49
Collection and monitoring of medical measures (eg, pain and blood pressure)	0.58	1.41
Reminders about the warning signs of stroke or TIA and what to do	—	0.96
Receiving encouraging or persuasive messages	0.01	0.94
Tools to manage your medication	0.13	0.65
Reminders (eg, appointments)	0.27	0.53
Tools to manage mental health	0.19	0.51
Supporting clinician-patient communication	0.3	—
Receiving instructions from clinicians (eg, text via app, SMS text message, and emails)	0.29	—
Receiving virtual *awards* as encouragement for achieving goals	0	0.33
Supporting clinician-patient communication (via messages from patients)	—	0.25

^a^TIA: transient ischemic attack.

^b^Data not available for this population.

#### Preferred Measures to Collect and Monitor

**Figure 4:**
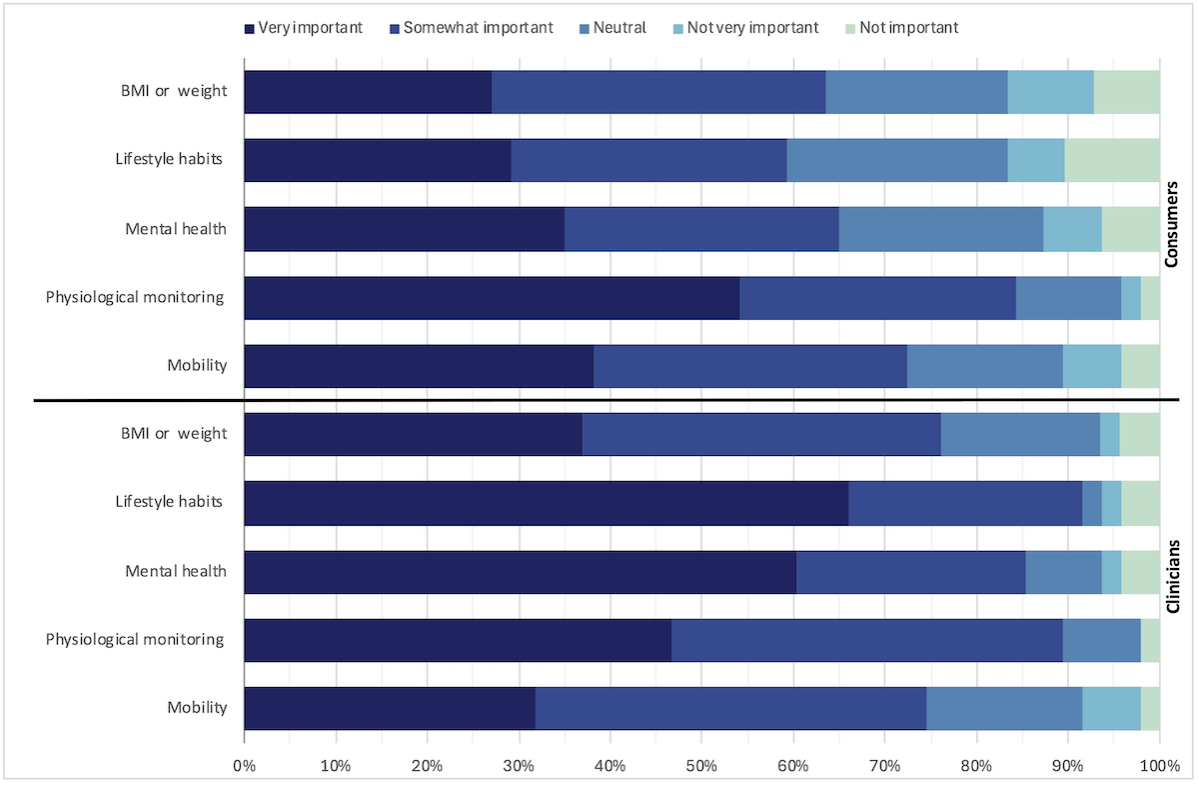
Consumers’ (n=96) and clinicians’ (n=48) rating of the types of measures that should be collected in a digital support program for people after stroke or transient ischemic attack.

Consumers rated physiological measures (eg, blood pressure, heart rate, and sleep quality), followed by mobility (eg, range of motion) and mental health measures (eg, stress and anxiety), as the top health and well-being indicators to monitor. Clinicians agreed on the importance of physiological and mental health measures but rated the collection of lifestyle data (eg, alcohol consumption, tobacco use, physical activity, diet, and nutrition) above all (Figure 4). Blood pressure, exercise, and heart rate were ranked by clinicians as the most important measures to collect via wearable and health monitoring devices (Table 4).

**Table 4:** Clinician participants’ ranking of measures that would provide value and utility in the management and secondary prevention of stroke. Participants ranked each function from 1 (most important) to 3.

Measurement	Ranking score
Blood pressure	1.76
Exercise	1.43
Electrocardiogram/heart rate/rhythm	1.29
Falls	0.60
Blood glucose	0.38
Body weight	0.31
Pain	0.29
Blood oxygen	0.1
Sleep	0.1
Range of motion	0.07
Body temperature	0.05

Most clinicians (46/49, 94%) also indicated the need for data monitoring more frequently than current practice, particularly related to waist circumference, dietary intake, and physical activity (Multimedia Appendix 3). Different preferences existed depending on the profession.

#### Perception of Digital Technology to Support Their Health

When asked about their perceptions of digital technology to manage and support their health goals after stroke or TIA, most consumers and clinicians agreed that a program that uses digital technology could be useful to monitor the consumers’ health and well-being (consumers: 78/97, 80%; clinicians: 40/42, 95%) and help them adapt to a new lifestyle (consumers: 76/99, 77%; clinicians: 38/42, 90%). More than 70% of consumers and clinicians also noted their intention to use the digital program as often as needed (consumers: 71/99, 72%; clinicians: 33/42, 78%) and discuss the data collected with their physicians (patients consumers: 79/99, 80%; clinicians: 36/42, 86%; Multimedia Appendices 4 and 5). Clinicians agreed they would review the data during consultations (26/42, 62%), at least once a week (10/42, 24%), or occasionally (3/42, 7%). Only 3 (all nurses) mentioning that they were too busy to review the data. When asked how they want to access the collected data, >97% (41/42) of clinicians noted their interest to access the data. Moreover, 52% (22/42) would like to access the data via a clinician web portal and a mobile app, 21% (9/42) would like to look at the data via the consumers’ app during consultations, 17% (7/42) would like to access the data via a clinician web portal, 5% (2/42) would like to access the data via a clinician app, and 2% (1/42) would like the data to be linked to the patients’ medical records. Most clinicians (35/42, 83%) expressed their intention to use the program to care for people after stroke or TIA.

### Stage 2: User Experience Workshops

#### Overview

A total of 7 consumers (male individuals: n=5, 71%) and 9 clinicians (female individuals: n=6, 67%) took part in the user experience workshops. In the design stage, 3 sessions (1 group and 2 individual sessions) were conducted with 5 consumers and 3 sessions (2 group and 1 individual sessions) were conducted with 6 clinicians. In the usability testing, a total of 4 individual sessions were conducted with consumers and 4 with clinicians. A total of 2 consumers and 2 clinicians took part in both the design workshops and usability testing (Table 5).

**Table 5:** Participant demographics of the user experience workshops.

Participant demographics			Consumers (n=7)		Clinicians (n=9)
**Sex, n (%)**					
	Male	5 (71)		3 (33)	
	Female	2 (29)		6 (67)	
Age (y), range			67-84		<29-49
**Workshops, n (%)**					
	Design	3 (43)		5 (56)	
	Usability testing	2 (29)		2 (22)	
	Both	2 (29)		2 (22)	
**Diagnosis, n (%)**					
	Stroke	4 (57)		—^a^	
	Transient ischemic attack	3 (43)		—	
**Profession or role, n (%)**					
	Registered nurse	—		2 (22)	
	Neurologist	—		1 (11)	
	Physiotherapist	—		2 (22)	
	Stroke Foundation representative	—		1 (11)	
	Stroke coordinator	—		2 (22)	
	Speech pathologist	—		1 (11)	

^a^Data not available for this population group.

#### Design Workshop

Qualitative analysis focused on four practical themes of user experience and preferences: (1) the consumers’ experience using mobile apps and wearable devices, (2) consumer and clinician perception of mHealth for secondary stroke prevention, (3) preferred functions, and (4) preferred health indicators for a digital program for secondary prevention of stroke. In these workshops the terms *patient* and *client* were used interchangeably by clinicians when referring to consumers.

##### Consumers’ Experience Using Mobile Apps and Wearable Devices

All consumer participants confirmed that they used digital technology regularly, including smartphones, tablets, and PCs, for activities such as banking, taking photographs, appointment reminders, phone calls, and taking notes. Most of them also used alarms for medication reminders. Many of them used older devices that were given to them by their children or grandchildren and mentioned that while they knew how to use them, they did not feel as comfortable as the younger populations or as they were before their strokes:

I’ve got my daughter’s old Apple phone. I learned it quickly. After stroke I managed it reasonably well still, but I cannot do everything on it, I don’t know how to do it.

Consumer 2

Most of them used apps to track their exercise, particularly their step counts. Only 1 participant reported using a smart watch (Samsung watch, Samsung Electronics Co Ltd) to track their exercise. No other health-related apps were reported, with consumers noting that they only used these apps when they were suggested by their treating clinicians.

##### Perception of mHealth for Secondary Stroke Prevention

Clinicians mentioned that regular contact with consumers was only provided while they were actively involved with their service (eg, rehabilitation therapy), with only annual monitoring provided after discharge. They noted that after discharge, self-monitoring was important, with some clinicians expressing their interest in a telehealth model of care where they could monitor consumers remotely (eg, via a clinician portal) and look at a summary of their data during clinical visits. They all agreed that providing tools to educate and empower consumers to self-manage and self-monitor their condition was important:

I feel like a lot of our role is around education...they also need to have the tools to enable them to...perform some of that self-monitoring where they are able to do so.

Clinician 1

All consumer participants supported the idea of a mobile app to help them manage their stroke or TIA.

##### Preferred Functions

Consumers noted that receiving alerts or early warnings of potential deteriorations and medication reminders were their main interests:

Medication alarm is my favorite...

Consumer 2

They also mentioned that the ability to record information on their phone to share with their treating clinicians would be relevant. They expressed particular interest in accessing a graph or a summary of their information to track their own progress. Most clinicians agreed that the collection of regular and accurate data to support self-monitoring and clinical management would be beneficial to track changes over time, particularly during the first weeks to months after discharge:

I can see its benefits outside the acute setting in the weeks and month following discharge.

Clinician 7

One consumer highlighted that their handwriting was becoming difficult to read and expressed the need for an app to replace handwritten notes to record information that needed to be shared with their physician.

When asked about the need for educational content, consumers agreed that they trusted their clinicians and hospital more than the information available in the web, so they were not interested in alternative sources of information. However, they were interested in having access to the information provided to them during their clinical visits, including the warning signs of stroke and updated information from their clinicians:

I trust the specialist who has kept me alive.

Consumer 1

Both clinicians and consumers agreed that the use of gamification techniques, such as virtual medals, was inappropriate:

Receiving virtual medals may be to a very, very slight few rewarding...but in general really not appropriate.

Clinician 6

##### Preferred Health Indicators to Collect via mHealth for Secondary Prevention

When talking about data collection, consumers agreed that blood pressure was the most relevant data they collected to manage their risk factors after stroke. Some of them collected it regularly, while others only if they felt unwell or if their medication changed. In this vein, clinicians agreed that consumer compliance was high, particularly in the context of the collection of blood pressure. However, they mentioned that lifestyle and exercise data were not routinely collected even if it was considered important because consumers’ reports were often unreliable:

A lot of this relies on self-reporting, and patients might under-report or overestimate the amount of exercise.

Clinician 4

Some clinicians mentioned that well-being and lifestyle data were regularly collected over the phone, with fatigue often coming up as relevant. Consumers echoed their interest to record and track their fatigue levels after stroke using a reliable scale.

Some clinicians also mentioned that social isolation was particularly important for people living in remote areas and noted that this was commonly measured visually (eg, signals of neglect, lack of access to medications, and disheveled appearance) as some consumers preferred not to talk about it:

Talking to clients, hoping that they are willing to talk about isolation and support. Some clients willingly and openly talk about it and others won’t.

Clinician 5

Few clinicians mentioned that electrocardiogram and waist circumference were not particularly important, while 1 participant noted that medication adherence and health care use history were relevant.

#### Usability Testing

##### Overview

Following the design workshops, wireframes and prototypes for the CAPS program were developed using 3 main technologies: a consumer-facing mobile *app* accessed via smartphones or tablets, a *smart watch*, and a *clinician web portal*. These prototypes were used to collect additional participant feedback during the user testing workshops. This section provides the main insights from both consumer and clinician to guide the final design of the CAPS platform.

##### Mobile App and Wearable Feedback and Insights

The app design needs to be simple, with a shallow architecture (without multiple screen levels) to reduce cognitive load. The use of pictures and icons was preferred over text. Labels and instructions should be clear, with large buttons and scalable font size. Some participants also suggested an option for the app to “speak” instruction and the ability to collect data through voice, for consumers whose reading or writing ability was affected:

[L]ove the fact that you’ve got the icons there, it’s really easy to see what each of the options are and represent.

Clinician 1

The home screen should prioritize the most important functions, with a daily check in presented as steps and with clear signs to mark its completion. Many participants expressed the need to view historical data points.

A “symptoms” screen that provides educational information about the early signs of stroke was suggested, but there were mixed opinions about the need to include any other educational content within the app.

The ability to add notes into a health journal was noted. Furthermore, some participants mentioned that using a camera to add pictures within the notes might be difficult for some users and expressed privacy concerns.

Participants also supported the inclusion of several data sources, including weight, blood glucose levels, alcohol and tobacco consumption, ECG, measures of social interaction and social support, and an option to add activities (eg, walking and swimming) and step counts using a smartphone if a smart watch was not available.

Finally, participants questioned the practicalities of collecting survey data via the smart watch, given the small form factor, but strongly supported the use of the smart watch for sensor-based data collection and reminders.

##### Clinician Portal

The clinician portal was designed to be used for onboarding and monitoring of consumers. In this vein, participants noted that the onboarding process should allow clinicians to record consumer’s consent and a set of secondary prevention goals. Data collection needs and alerts, when data are out of bounds, should be customizable via the portal, so that they are personalized to consumers. There was also a suggestion for clinicians to manage medication reminders via the clinician portal, providing consumers with the ability to track their compliance via a “tick box” within the app:

[P]erhaps have a little reminder to say, okay, it’s time to take your medication. This might be something that the clinician sets up.

Clinician 3

For consumer monitoring, some participants requested a customizable dashboard view with traffic light system (red, orange, and green) to quickly flag individuals that need prompt follow-up and facilitate monitoring. They also highlighted the importance of an “export function” that would allow them to download (as pdf, csv, etc), print out, and share a summary of the data when needed.

### The CAPS Platform

#### Overview

**Figure 5:**
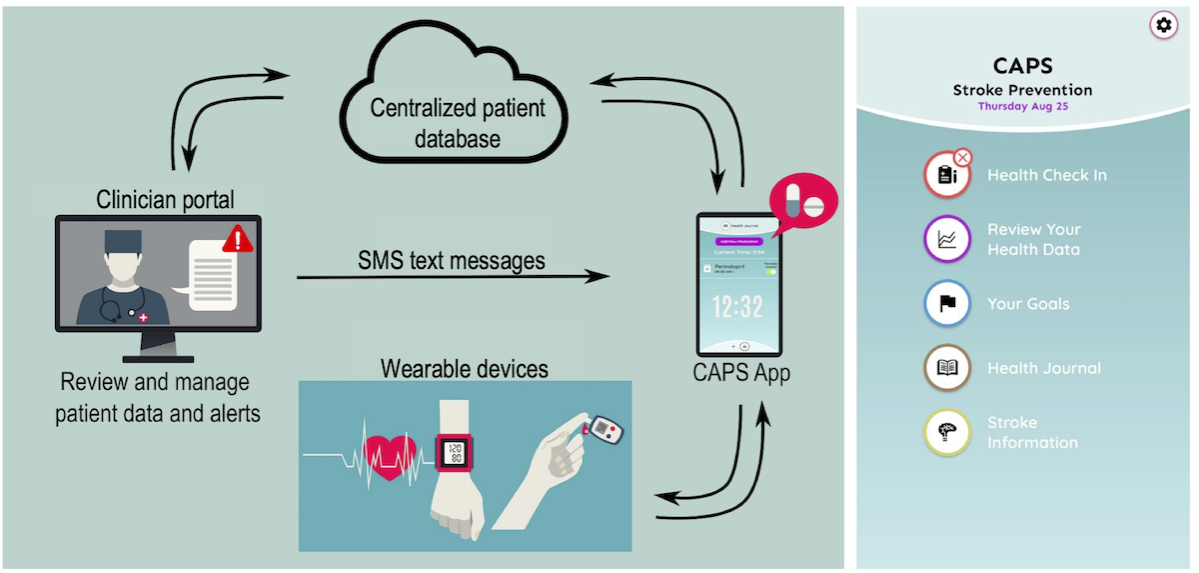
The Care Assistant and support Program for people who have experienced a Stroke or transient ischemic attack (CAPS) platform model (left) and the CAPS mobile app home screen (right).

The final CAPS platform (Figure 5) was designed using the evidence from the co-design stages and consists of a mobile app (available in iOS and Android versions), supported by both smartphones and tablets, that integrates wearable activity trackers and smart devices (Fitbit watches and fitness trackers, Oura ring, or Apple Watch) and a clinician web portal (Portal) that includes an electronic message system (Message system). All data collected by the wearable devices are synchronized via Bluetooth to the app and uploaded to the Portal.

#### Mobile app

**Figure 6:**
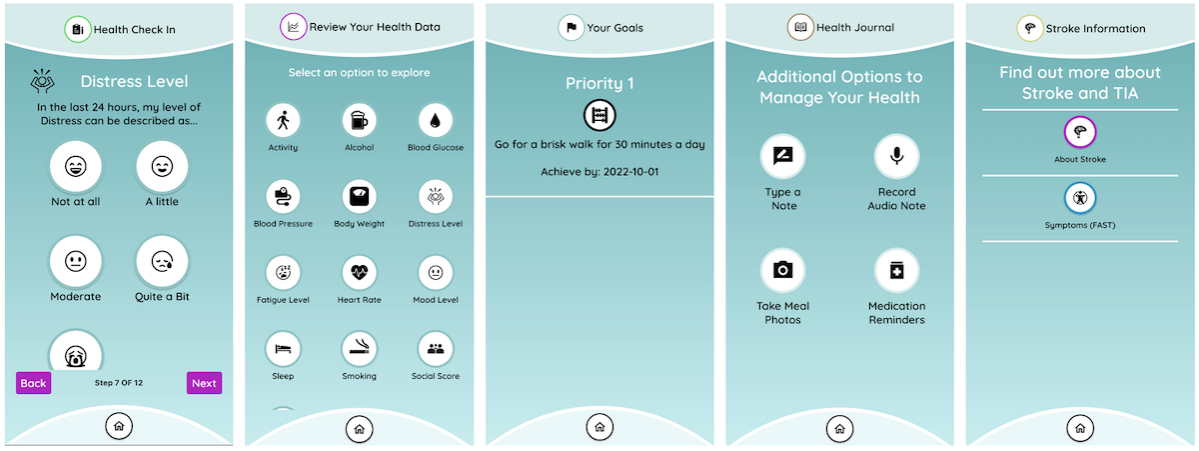
Key features of the CAPS (Care Assistant and support Program for people who have experienced a Stroke or transient ischemic attack) mobile app. From left to right: health check in (distress level question 7 of 12), review health data, goals, health journal, and stroke information.

The app is designed for use by consumers daily. Figure 6 highlights the key features.

##### Health Check In

A daily check in of self-recorded data, including activities, blood pressure, distress, and other relevant health measures customized by clinicians during onboarding (Table 6). Most of the questions and answers included in this section were informed by the literature or clinical expert advice, including those related to blood pressure, blood cholesterol, blood glucose, body weight, alcohol and cigarette consumption [8], mood [45,46], distress [46], fatigue [47], social connection [48-50], social support [48-50], and social isolation [51]. The daily health check in was designed to be easy to understand and takes <5 minutes to complete.

**Table 6:** Health check-in questions available in the CAPS (Care Assistant and support Program for people who have experienced a Stroke or transient ischemic attack) app.

Item	Question	Answer options
Activities	Select activities done in the last day	Walking, swimming, cycling,..., other exercise
Alcohol	Number of alcoholic drinks in the last day	Discrete numbers
Blood glucose	Current blood glucose (mmol)	Discrete numbers
Blood pressure	Systolic and diastolic value	Discrete numbers
Body weight	Current weight (kg)	Discrete numbers
Tobacco	Number of cigarettes consumed in the last day	Discrete numbers
Distress	In the last 24 hours, my level of distress can be described as...	Not at all, a little, moderate, quite a bit, and extremely
Fatigue	My fatigue level is...	Not fatigued at all, a little fatigued, moderately fatigued, very fatigued, total fatigue, and exhaustion
Mood	Today I feel...	Happy, somewhat happy, neutral, somewhat sad, and sad
Social connection	In the last 24 hours, I was satisfied with the amount of time I spent talking or meeting with people who do not live with me	Strongly agree, agree, neutral, disagree, and strongly disagree
Social support	In the last 24 hours, support from family, friends, health workers, or others was available when I needed it	Strongly agree, agree, neutral, disagree, and strongly disagree

##### Review Health Data

Reviewing and monitoring of historical self-recorded data and data collected by the wearable devices (eg, heart rate, physical activity, and sleep). Data are graphically displayed.

##### Goals

Secondary prevention SMART (specific, measurable, achievable, relevant, and time-bound) goals [52,53] developed by clinicians with consumers are captured within the app.

##### Health Journal

Notes (text and audio) and photos can be taken and recorded for anything that is helpful or relevant to consumers. These data are kept private within the mobile device and are not uploaded to the clinician portal. Medication reminders can be set up to prompt consumers to take their medications.

##### Stroke Information

In addition to a symptom section based on the FAST (facial drooping, arm weakness, speech difficulties, and time to call emergency) acronym, web links related to practical information of stroke prevention and healthy living are provided.

##### Settings (Top Right Gear)

Allows consumers to manage SMS text messages, notifications, and font size.

#### Portal

The portal was designed for use by clinicians and their assistants during onboarding and data monitoring. Key features are described below.

##### Onboarding and Setup

Health measures required to be collected by consumers, thresholds for health measure alerts, and secondary prevention goals can be set and captured based on the characteristics of individual consumers.

##### Consumer Dashboard

**Figure 7:**
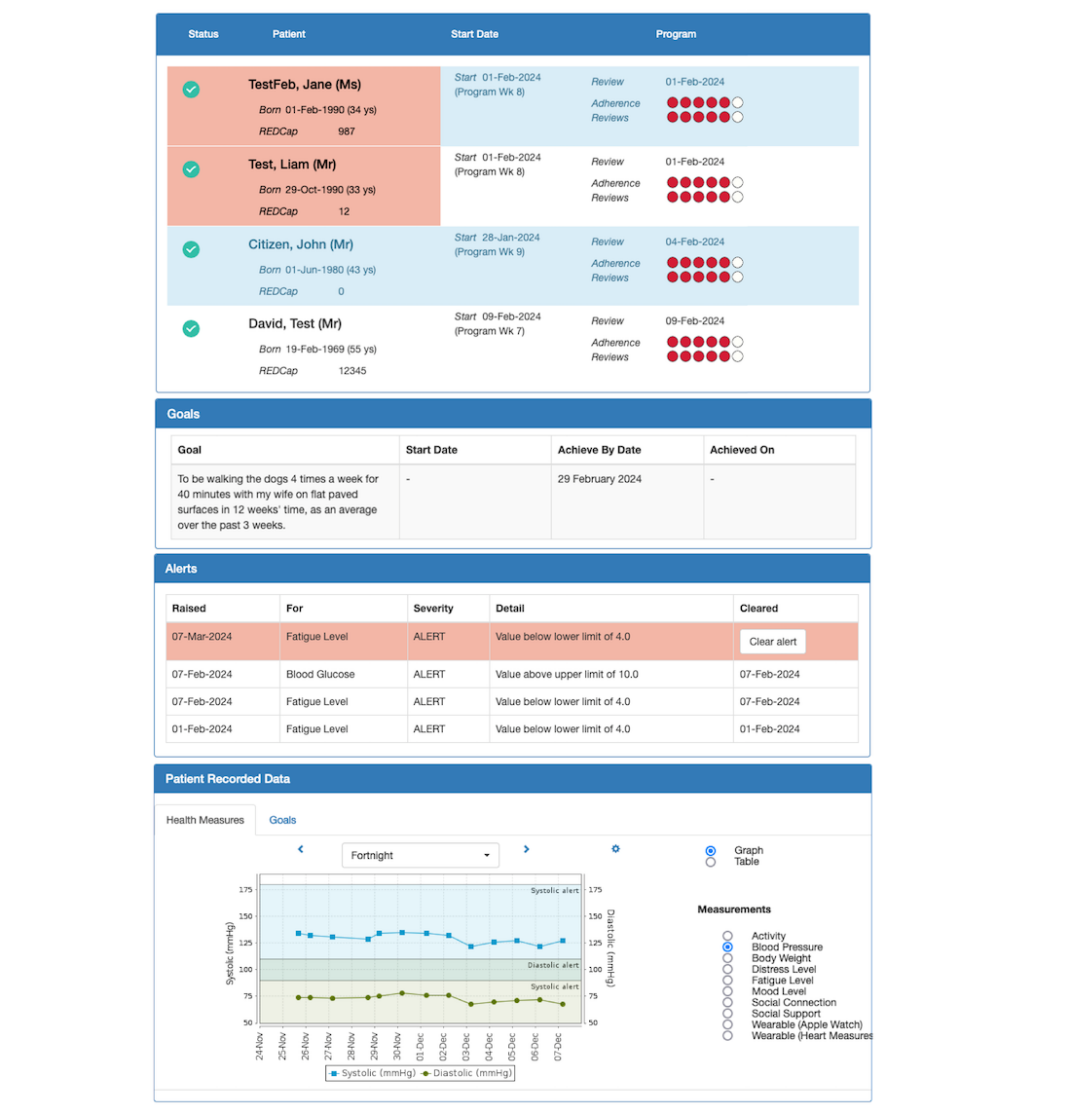
The Care Assistant and support Program for people who have experienced a Stroke or transient ischemic attack clinician portal. From top to bottom: consumer dashboard home screen, participant goals screen, alerts screen, and patient recorded data screen (blood pressure graph).

Presents an “at a glance” summary of all consumers, including goals and alert highlights if a consumer’s health measures are out of normal range. Data can be downloaded into CSV or PDF files (Figure 7).

##### Consumer Record Details

Presents a summary of a consumer’s personal details, collected data, and goals. Longitudinal data gathered via the consumer app and wearable can be displayed in graph or table formats.

##### Message System

Clinicians can assign personalized electronic messages to be automatically sent to consumers, aligned with their secondary prevention goals.

## Discussion

### Principal Findings

Effective management of long-term lifestyle behavior changes following discharge after stroke or TIA is essential for secondary prevention. This paper presents the co-design of a novel, multicomponent, clinician-facilitated, patient-centered digital program to support secondary prevention of stroke, which is now ready for feasibility testing with clinicians and people with lived experience of stroke or TIA.

The development of CAPS followed a multistaged co-designed process that considered the needs and preferences of both consumers and clinicians involved in stroke management. The user needs surveys and user experience workshops confirmed the need for a program to support self-management of lifestyle changes after discharge, with most participants managing their lifestyle changes without a rehabilitation program (108/112, 96.4%) or the support of a health professional (71/112, 63.4%).

When considering a digital program for secondary prevention of stroke, participants noted particular interest on supporting an individual’s ability to adapt to a new lifestyle (eg, physical activity), the collection and monitor of lifestyle (eg, steps and sleep) and physiological measures (eg, blood pressure and heart rate), and receiving alerts about potential deterioration in their health. This was expected, given that clinical practice guidelines prioritize the control of blood pressure and atrial fibrillation for stroke prevention. In addition, lifestyle modifications to reduce individual risk for cardiovascular disease or stroke are key performance quality indicators of care; however, >25% of patients are not provided discharge education targeting their personal cardiovascular risk factors, indicating the need to address this clinical practice gap [54].

Surveys also highlighted the clinical need for more regular access to consumers’ data. These results align with the global trend to incorporate emerging technologies, such as mobile apps, sensors, and wearable devices, in health applications that target early identification and tailored treatment of health conditions [55].

With most consumer participants (91/112, 81.2%) reporting the use of smartphones regularly and most clinicians (33/42, 78%) and consumers (71/99, 72%) reporting their intention to use the program, the potential for an mHealth program to support secondary prevention for stroke was strong. Participants also believed a mobile app would fit well with their routine and has the potential to improve their recovery and quality of life.

When considering the use of a mobile app in secondary prevention of stroke, participants highlighted the importance of accuracy in the collected data. The need to provide clinicians access to the collected data was also rated highly by consumers. This aligns with previous mobile health evaluations that have shown the value of remote monitoring in a patient’s engagement and motivation [34]. The inclusion of general information about stroke or TIA treatment and prevention that consumers could access in their own time was suggested by clinicians. Consumers, by contrast, preferred to receive their educational content directly from their hospital or treating clinicians but supported the inclusion of the information provided to them during their clinical visits (including the warning signs of stroke) into the app. The inclusion of a function to provide medication reminders was also requested by some consumers.

### The CAPS Platform

On the basis of the information collected during the user needs surveys and the design workshops, a prototype of a consumer-facing mobile app accessed via smartphones or tablets, a smart watch, and a clinician web portal were developed and user tested with both consumer and clinician participants. During this stage, participants noted the need for the app to be easy to use, with large font size, large buttons, and iconography to facilitate user engagement. The ability for the app to “speak” instruction and collect data through voice was also noted for consumers whose reading or writing ability is affected. They also questioned the need to share personal notes or recordings and photographs with clinicians, due to privacy concerns.

Following the user testing stage, a mobile app and clinician portal were developed using a user-centered design that includes a single check-in stage that is clear, simple, and can be tailored (during onboarding) based on consumer needs. While the questions included in the check-in stage were informed by the literature, their correlation with standard tools (eg, the Depression Anxiety Stress Scale-21 item [56] and the Duke Social Support Index [48]) will be evaluated in future research. The collected data can be displayed on a graph (on both the mobile app and the clinician portal) or can be downloaded and printed to be used more effectively by both consumers and clinicians. Informed by the literature [57,58], the program also allows clinicians to send SMS text messages to participants with relevant information to support their secondary prevention goals. One-way SMS text messages were preferred over app notifications due to the increased ability to ensure the delivery of the messages. The inclusion of gamification techniques was discarded, as it was considered inappropriate for this population.

An important consideration was the inclusion of a medication reminder function. While the need for this function was supported by clinicians and consumers, there was no agreement on the safety and practical implementation. After detailed consideration, it was decided to provide consumers with the ability to include their own medication reminders as needed with notifications in both, the app and the wearable device, but not the ability to track compliance due to increased risk on medication errors. The clinician portal was developed to be accessed via a website, as was indicated by clinician preferences (29/42, 69%).

Clinicians and consumers highly rated the importance of collecting data on mental health (eg, stress, anxiety, and depression). Psychological distress, which incorporates elevated symptoms of depression, anxiety, and psychological stress [59], occurs frequently in people living with stroke [60]. Poststroke emotional distress is also associated with poor clinical outcomes, including poor medication adherence, poor quality of life, poor engagement with rehabilitation, and increased mortality [45]. Hence, it was decided, that the daily check in should capture both mood and distress to enable analysis and correlation with validated tools, which will provide evidence for the most appropriate item to remain in the mobile app. Emoji icons to represent mood and distress were selected guided by previous work [45]. There was also significant clinical support for the collection of fatigue and social isolation data, which has become particularly relevant since the COVID-19 pandemic.

The CAPS program aligns with the World Health Organization’s recommendations for digital health interventions that support equitable access to quality health services that strengthen and scale up health promotion and disease prevention strategies [61]. A strength of the CAPS programs is the co-design approach and process to ensure this digital health strategy addresses the needs of people with lived experience of stroke or TIA and clinicians. We are now ready to take the program to feasibility testing to ensure it is something that would be of benefit to implement into clinical practice as a secondary prevention stroke program.

### Limitations

While AuSCR facilitated access to registrants across Australia and provided access to a broad range of people with stroke (72/112, 64.3%) and TIA with wide age range (aged 40-98 years), it was difficult to access consumers with a recent experience of stroke or TIA (ie, within the last 6 months). Future work should explore alternative recruitment strategies to access those consumers.

While >50 clinicians answered all survey questions, including 13 medical doctors (eg, a neurologist, a cardiologist, and rehabilitation clinicians), we did not receive any responses from general practitioners. Lack of representation from primary health care needs to be addressed in future stages of the project, including feasibility testing and clinical evaluations. In addition, due to our recruitment methods, the clinicians who opted to participate were likely to be interested in the use of digital health to improve care for patients and may not have been fully representative of the greater stroke health care community.

### Comparison With Previous Work

There is currently little information in the literature for evidence-based and available digital health platforms specifically designed to support people living with stroke. However, there is increasing research in how mobile apps can be used to support people living with stroke. They have been used to facilitate the delivery of interventions promoting increased physical activity [62,63], blood pressure control [62,63], medication adherence [64,65], and stroke awareness [66,67]. Additional intervention components that have been used to supplement the mobile app include SMS text messages, gamification, GPS tracking, and pedometers, often with remote monitoring capabilities for clinicians [63,64,68,69].

While existing digital health apps incorporate various digital components, the CAPS program is unique to its comparators due to combining 4 technological components (mobile app, wearable device, clinician portal, and SMS text messages developed for a self-management support package for people who experienced a stroke) with standardized patient-focused secondary prevention goal setting [30]. This promotes congruity in perception of care for patients and clinicians, better supporting lifestyle behavior changes and goal attainment. The technology facilitates both active and passive data collection, with remote access to the data collected and the ability to respond to alerts for measurements outside clinical guidelines. The secondary prevention goals developed with trained personnel support patients to control and reduce risk factors, assisted by SMS text messages developed to promote behavior change.

### Conclusions

We have outlined the detailed co-design of the CAPS platform that aims to support secondary prevention of stroke via goal attainment and tailored mHealth strategies. Need, acceptability, and intention to use the digital program were supported by consumers and clinicians. Feasibility testing is currently in process. The password-protected app is available for download via the Android Play Store and the Apple Store.

## Appendix

Multimedia Appendix 1 Consumer survey.

Multimedia Appendix 2 Clinician survey.

Multimedia Appendix 3 Participants’ rated need to access data more frequently than current practice (n=49).

Multimedia Appendix 4 Total consumer participants’ perception on the introduction of a new support program that used digital technologies for people who had experienced a stroke or transient ischemic attack (n=105).

Multimedia Appendix 5 Clinician participants’ perception on the introduction of a new support program that used digital technologies for people who had experienced a stroke or transient ischemic attack (n=42).

## Abbreviations

AuSCR: Australian Stroke Clinical Registry
CAPS: Care Assistant and support Program for people after Stroke or transient ischemic attack
FAST: facial drooping, arm weakness, speech difficulties, and time to call emergency
mHealth: mobile health
REDCap: Research Electronic Data Capture
SMART: specific, measurable, achievable, relevant, and time-bound
TIA: transient ischemic attack
