# Supplementary material for: Multicomponent Support Program for Secondary Prevention of Stroke Using Digital Health Technology: Co-Design Study With People Living With Stroke or Transient Ischemic Attack
Source: J Med Internet Res. 2024 Aug 22;26:e54604. doi: 10.2196/54604 (PMC11377903; doi:10.2196/54604)
Supplement: Multimedia Appendix 1 [file jmir_v26i1e54604_app1.pdf]

Survey on digital health technologies used by people living with stroke or Transient Ischaemic Attack (TIA)

Postcode of your residential address:_________

Demographics

1. What is your Date of Birth (DD/MM/YYYY) _________

2. Sex

☐Male ☐Female ☐Prefer not to disclose ☐Other (please specify) __________________

3. What is your highest level of education?

☐Less than Year 12 or equivalent

☐Year 12 or equivalent

☐Vocational qualification

☐Associate diploma

☐Undergraduate diploma

☐Bachelor degree (including honours)

☐Postgraduate diploma

☐Master's degree

☐Doctorate

4. Do you primarily live alone or with others?

☐Alone ☐With others

If you live with others, what is your primary living arrangement? (choose all that apply)

☐With partner ☐With children ☐With other family ☐With others, not family ☐Other situation (please specify)_____________

Medical history

5. When was your most recent stroke or TIA (Transient Ischemic Attack) onset/diagnosed? (mm/yyyy) ______________________

6. Was this a stroke or TIA? ☐ Stroke ☐ TIA ☐ Don't know

7. Was this your first stroke/TIA? ☐ No ☐ Yes (If Yes, please go to question 10)

8. If this was not your first stroke, when was your first stroke or TIA onset/diagnosed? (mm/yyyy) _______________________

9. Was this a stroke or TIA? ☐ Stroke ☐ TIA ☐ Don't know

10. Do you have any difficulties with thinking or concentrating since your first stroke/TIA?

    ☐ Yes (a lot) ☐ Yes (sometimes) ☐ No (no difficulties)

11. Are you currently participating in a stroke/TIA rehabilitation program at clinics or hospitals?

    ☐Yes ☐No

12. Are you currently receiving a home rehabilitation program?

    ☐Yes ☐No

13. Do you currently see a health professional (such as specialist doctor, physiotherapist, dietician) to support your recovery or ongoing management after stroke?

    ☐Yes ☐No

14. Do you currently take medications to manage symptoms of your stroke or reduce your risk of a future stroke (e.g., blood pressure lowering, cholesterol-lowering, blood-thinning medication)?

    ☐Yes ☐No

Part 1. Experience using **mobile apps**, and preferred functions for a new digital support program

15. Do you use any mobile apps?(A mobile app is a type of application software designed to run on a mobile device, such as a smartphone or tablet computer)

    ☐Not applicable (I do not have a smartphone or tablet device)

    ☐Yes

    ☐No

    If you selected “Not applicable” or “No”, please go to question 22.

16. Please indicate which of the following devices you use to run your mobile apps? (tick all that apply)

    ☐Apple iPhone

    ☐Smartphone running Android operating system (e.g., Samsung)

    ☐Apple iPad

    ☐Tablet running Android operating system

    ☐Tablet running Microsoft Windows operating system

    ☐Tablet running other operating systems

    ☐Other (please specify) ______

17. Do you need help from another person to use the device(s) above?

    ☐Yes ☐No

18. How many mobile apps do you use regularly (at least once a week)?

    ☐None ☐<5 ☐6-10 ☐>10

19. How many **mobile apps** for managing your health and wellbeing do you use regularly (at least once a week)?

☐None ☐<5 ☐6-10 ☐>10

If you selected “None”, please go to question 22.

20. Which health conditions or purposes do you use **mobile apps** for? (check all that apply)

☐ Lifestyle support (e.g., smoking cessation, dietary intake, weight reduction)

☐Exercise tracking (e.g., step counting, workout management)

☐Managing specific medical disease conditions (please specify the condition) ______

☐Medication management such as reminder to take medications or fill scripts

☐Mental health support (e.g., anxiety, depression, mindfulness, meditation)

☐Other (please specify)______

21. How did you find out about the **mobile apps** for managing health condition and wellbeing? (check all that apply)

☐App associated with a tracking device (e.g., Fitbit, Garmin)

☐Pre-installed on my phone

☐Recommended by my friend, family member

☐Recommended by someone with a similar health condition

☐Recommended or prescribed by my doctor or other health care provider

☐Searching App store

☐Searching internet

☐Other (please specify) ____

22. If a digital support program were available that was designed specifically for a person living with stroke/TIA, how important are the following types of health and wellbeing functions to you? Please also rank the **top three most important** functions from the list, from 1 (most important) to 3.

**Functions**	**How important?** (Please tick one box indicate your rating of importance)					**Rank the top three most important**
	**Not important at all**	**Not very important**	**Neutral**	**Somewhat important**	**Very important**	
Alerts about potential deterioration in your health or stroke/TIA risk	1	2	3	4	5	
Collection and monitoring lifestyle measure (e.g., steps, sleep)	1	2	3	4	5	
Collection and monitoring of medical measures (e.g., pain, blood pressure)	1	2	3	4	5	
Communication with clinicians (audio and video)	1	2	3	4	5	
Educational information in general about stroke/TIA treatment and prevention	1	2	3	4	5	
Receiving instructions from clinicians (e.g., text via app, phone SMS, emails)	1	2	3	4	5	
Receiving encouraging messages	1	2	3	4	5	
Receiving virtual "awards" as encouragement for achieving goals	1	2	3	4	5	
Reminders (e.g., appointments)	1	2	3	4	5	
Tools to manage new lifestyle, (e.g., exercises, diet, alcohol, smoke)	1	2	3	4	5	
Tools to manage mental health	1	2	3	4	5	
Tools to manage your medication	1	2	3	4	5	
Other (please specify) _____	1	2	3	4	5	

23. If a digital health support program were available to assist your current stroke/TIA program and care, how important are the following types of measures would you want to be included? Please also rank the **top three most important** measures from the list, from 1 (most important) to 3.

**Measures**	**How important?** (Please tick one box to indicate your rating of importance for each measure)					**Rank the top three most important**
	**Not important at all**	**Not very important**	**Neutral**	**Somewhat important**	**Very important**	
Body mass index, waist circumference or weight	1	2	3	4	5	
Lifestyle habits such as alcohol consumption, tobacco use, physical activity, diet and nutrition	1	2	3	4	5	
Mental health, including stress, anxiety, depression management	1	2	3	4	5	
Physiological monitoring such as sleep, heart rate, blood pressure	1	2	3	4	5	
Mobility including pain and range of movement	1	2	3	4	5	
Other (please specify) _______	1	2	3	4	5	

Part 2. Experience of using **wearables and health monitoring devices**, and preferred health indicators for secondary prevention

24. Which of the following devices do you own or have access to? And if you have access to the devices, how often did you use them in the past months?

**Devices**	**Do you own or have access to this device?**		**If you own or have access to this device, in the past months, how often did you use the device?**				
Activity tracker (e.g., Fitbit)	Yes	No	Never	Occasionally	1-3 times/week	4-6 times/week	Daily
Smart watch (e.g., Apple Watch)	Yes	No	Never	Occasionally	1-3 times/week	4-6 times/week	Daily
Blood glucose monitor	Yes	No	Never	Occasionally	1-3 times/week	4-6 times/week	Daily
Blood oxygen monitor (e.g., pulse oximeter)	Yes	No	Never	Occasionally	1-3 times/week	4-6 times/week	Daily
Blood pressure monitor	Yes	No	Never	Occasionally	1-3 times/week	4-6 times/week	Daily
Device for detecting falls	Yes	No	Never	Occasionally	1-3 times/week	4-6 times/week	Daily
Device for managing pain	Yes	No	Never	Occasionally	1-3 times/week	4-6 times/week	Daily
Device for tracking range of motion (e.g., arm, leg)	Yes	No	Never	Occasionally	1-3 times/week	4-6 times/week	Daily
Electrocardiogram (ECG) monitor (e.g., Polar Band)	Yes	No	Never	Occasionally	1-3 times/week	4-6 times/week	Daily
Heart rate monitor	Yes	No	Never	Occasionally	1-3 times/week	4-6 times/week	Daily
Sleep monitor	Yes	No	Never	Occasionally	1-3 times/week	4-6 times/week	Daily
Thermometer	Yes	No	Never	Occasionally	1-3 times/week	4-6 times/week	Daily
Weight scale	Yes	No	Never	Occasionally	1-3 times/week	4-6 times/week	Daily
Other (please specify) ____________________	Yes	No	Never	Occasionally	1-3 times/week	4-6 times/week	Daily

25. How did you choose your **wearables and health monitoring devices**? (check all that apply)

- ☐N/A. I do not have any devices
- ☐Recommended or prescribed by my doctor or other care provider
- ☐Recommended by someone with similar health condition
- ☐Recommended by my friend or family member
- ☐Searching internet
- ☐I do not remember
- ☐Other (please specify) _______

26. If a digital health support program were available, which can integrate **wearable and health monitoring equipment** to assist you to manage your health after stroke/TIA, how important would monitoring the following health and wellbeing indicators be to you? Please also rank the **top three most important** functions from the list, from 1 (most important) to 3.

**Items**	**How important?**<br><br>(Please tick one box to indicate your rating of importance for each item).					**Rank the top three most important**
	**Not important at all**	**Not very important**	**Neutral**	**Somewhat important**	**Very important**	
Alcohol consumption	1	2	3	4	5	
Blood glucose	1	2	3	4	5	
Blood oxygen	1	2	3	4	5	
Blood pressure	1	2	3	4	5	
Dietary intake	1	2	3	4	5	
Electrocardiogram (ECG) monitor (e.g., Polar Band)	1	2	3	4	5	
Heart rate or rhythm	1	2	3	4	5	
Pain	1	2	3	4	5	
Physical activities (e.g., steps, stairs)	1	2	3	4	5	
Intensive physical activity (e.g., swim, gym, yoga, bicycle)	1	2	3	4	5	
Range of motion of arm/leg	1	2	3	4	5	
Sleep quality	1	2	3	4	5	
Tobacco use	1	2	3	4	5	
Weight	1	2	3	4	5	
Other (please specify)_____________	1	2	3	4	5	

Part 3 Attitude towards digital health technology for secondary prevention and support for stroke and TIA patients

27. When thinking about using digital health technology to support your care after stroke or TIA, how **important** are the following considerations to you? Please circle/tick one box to indicate your rating of importance for each consideration below.

**Items**	**Not important at all**	**Not very important**	**Neutral**	**Somewhat important**	**Very important**
Easy to use	1	2	3	4	5
Easy to setup and connect	1	2	3	4	5
Easy to integrate with my other devices and technologies	1	2	3	4	5
Availability of technical support	1	2	3	4	5
Accuracy of the data collected	1	2	3	4	5
Privacy of my data	1	2	3	4	5
Privacy of my activities	1	2	3	4	5
Sharing of my data with family member/carer	1	2	3	4	5
Tailored to address my care needs	1	2	3	4	5
Enjoyable to use	1	2	3	4	5
Cost (affordable)	1	2	3	4	5
Getting alert if abnormal readings are detected	1	2	3	4	5
My clinicians can access and monitor my data	1	2	3	4	5

28. If a **new support program which used digital technology was available now** for people who had experienced a stroke/TIA, please rate how much you agree with each of the following statements? Please circle/tick one box to indicate your agreement with each statement below.

Items	Answers				
	**Strongly disagree**	**Disagree**	**Neutral**	**Agree**	**Strongly agree**
Perceived usefulness					
Using a digital program could improve my quality of life	1	2	3	4	5
Using a digital program could improve my recovery after stroke or TIA	1	2	3	4	5
Using a digital program could make the management of my health easier	1	2	3	4	5
Using a digital program could help communicate with my doctor	1	2	3	4	5
Using a digital program could be useful to monitor my health and wellbeing	1	2	3	4	5
Using a digital program could help me adapt to a new lifestyle for secondary prevention of stroke/TIA	1	2	3	4	5
A digital program could help me follow clinicians' directions	1	2	3	4	5
Perceived ease of use					
Learning to use a digital program would be easy for me	1	2	3	4	5
I would be able to use a digital program without much effort	1	2	3	4	5
It would be easy for me to become skilful in using a digital program	1	2	3	4	5
Social influence					
My friends and family would expect me to use a digital program	1	2	3	4	5
My doctor would be supportive of me using a digital program	1	2	3	4	5

	Strongly disagree	Disagree	Neutral	Agree	Strongly agree
Intention to use					
I would intend to use the digital program in my management of stroke/TIA	1	2	3	4	5
I would intend to use the digital program as often as needed	1	2	3	4	5
I would intend to use the digital program daily	1	2	3	4	5
I would intend to discuss the data collected in the digital program with my doctor when I have face-to-face contact with them	1	2	3	4	5
Personal innovativeness					
If I hear about a new technology to manage my health, I usually look for ways to experiment with it	1	2	3	4	5
Among my peers, I am usually the first to try out new technologies for health care	1	2	3	4	5
Self-efficacy					
Using a digital program would be somewhat intimidating for me	1	2	3	4	5
I would be hesitant to use a digital program for fear of making mistakes that I cannot correct	1	2	3	4	5
I would feel apprehensive about using a digital program	1	2	3	4	5
Compatibility					
A digital health program would fit in well with my lifestyle	1	2	3	4	5
A digital health program would be compatible with most aspects of my life	1	2	3	4	5

29. Do you have any comments or suggestions for the design features for a digital health support program for people living with stroke and TIA?

____

____

____

30. Would you be willing to be contacted to be further involved in the study, e.g., to participate in workshops to design and test the features of the digital health support program for people living with stroke or TIA?

☐ Yes ☐ No

If yes, can you provide your best contact details?

Name:

Contact number:

Email:

Thank you for your time in completing this survey.
