# Supplementary material for: Multicomponent Support Program for Secondary Prevention of Stroke Using Digital Health Technology: Co-Design Study With People Living With Stroke or Transient Ischemic Attack
Source: J Med Internet Res. 2024 Aug 22;26:e54604. doi: 10.2196/54604 (PMC11377903; doi:10.2196/54604)
Supplement: Multimedia Appendix 2 [file jmir_v26i1e54604_app2.pdf]

Survey on digital health technologies used by clinicians to support people after stroke or Transient Ischaemic Attack (TIA)

Demographics

1. Age (years)

   ☐ <=29 ☐ 30-39 ☐ 40-49 ☐ 50-59 ☐ 60-69 ☐ 70-79 ☐ >=80

2. Sex

   ☐ Male ☐ Female ☐ Prefer not to disclose ☐ Other (Please specify) _____________

3. Profession (please tick)

   ☐ General practitioner

   ☐ Medical doctor (specialist: neurology, cardiology, rehabilitation, general medicine)

   ☐ Occupational therapist

   ☐ Physiotherapist

   ☐ Registered nurse, if ticked please define role: ☐ Advanced Practice Nurse ☐ Enrolled Nurse ☐ Registered Nurse

   ☐ Speech pathologist

   ☐ Social worker

   ☐ Other (please specify) ________

4. Type of clinical setting/area where you practice the most:

   ☐ Acute hospital ☐ Community-based service ☐ Primary healthcare ☐ Rehabilitation ☐ Other (Please specify) _____________

5. Years since completed entry-level clinical training: ________(years) ☐ Not applicable

6. Approximately, how many hours per week do you typically spend providing care to patients? ________(hours)

   What % of this time do you typically spend on providing care to people with stroke/TIA? _________ (%)

7. Approximately, how many people with stroke/TIA do you treat per week? _______

Part 1. Health measurements for the management of people with stroke and TIA

8. For effective management and secondary prevention of stroke or TIA, how important is it to you to collect the following measures from your patients, and how often are they currently collected in your service? What additional health measures would enhance the clinical care you provide to individuals with stroke or TIA? (Tick the best answer):

**Activity Items**	**How important is it to collect?**<br>*Not important at all (1),*<br>*Not very important (2),*<br>*Neutral (3),*<br>*Somewhat important (4),*<br>*Very important (5)*					**How often do you have access to this data currently?**<br>*daily, weekly, monthly, occasionally, never*					**How often you would like to access to this data in an ideal world?**<br>*daily, weekly, monthly, occasionally, never*				
Anthropometrics															
Weight	1	2	3	4	5	daily	weekly	monthly	occasionally	never	daily	weekly	monthly	occasionally	never
Body Mass Index	1	2	3	4	5	daily	weekly	monthly	occasionally	never	daily	weekly	monthly	occasionally	never
Waist circumference	1	2	3	4	5	daily	weekly	monthly	occasionally	never	daily	weekly	monthly	occasionally	never
Lifestyle															
Alcohol consumption	1	2	3	4	5	daily	Weekly<br>2	monthly	occasionally	Never<br>-5	daily	weekly	monthly	occasionally	never
Dietary intake (e.g., calories, sodium, saturated fat)	1	2	3	4	5	daily	weekly	monthly	occasionally	never	daily	weekly	monthly	occasionally	never
Tobacco use	1	2	3	4	5	daily	weekly	monthly	occasionally	never	daily	weekly	monthly	occasionally	never
Sedentary lifestyle (less than 150 minutes moderate physical activity per week)	1	2	3	4	5	daily	weekly	monthly	occasionally	never	daily	weekly	monthly	occasionally	never
Physical activity															
Physical activity (e.g., steps, stairs)	1	2	3	4	5	daily	weekly	monthly	occasionally	never	daily	weekly	monthly	occasionally	never
More intensive physical activity (e.g., running, swimming, gym, bicycle)	1	2	3	4	5	daily	weekly	monthly	occasionally	never	daily	weekly	monthly	occasionally	never

Physiological monitoring															
Sleep (hours per night/quality)	1	2	3	4	5	daily	weekly	monthly	occasionally	never	daily	weekly	monthly	occasionally	never
Continuous heart rate	1	2	3	4	5	daily	weekly	monthly	occasionally	never	daily	weekly	monthly	occasionally	never
Resting heart rate	1	2	3	4	5	daily	weekly	monthly	occasionally	never	daily	weekly	monthly	occasionally	never
Temperature	1	2	3	4	5	daily	weekly	monthly	occasionally	never	daily	weekly	monthly	occasionally	never
Blood pressure	1	2	3	4	5	daily	weekly	monthly	occasionally	never	daily	weekly	monthly	occasionally	never
Blood oxygen	1	2	3	4	5	daily	weekly	monthly	occasionally	never	daily	weekly	monthly	occasionally	never
Blood glucose	1	2	3	4	5	daily	weekly	monthly	occasionally	never	daily	weekly	monthly	occasionally	never
ECG	1	2	3	4	5	daily	weekly	monthly	occasionally	never	daily	weekly	monthly	occasionally	never
Mobility															
Number of falls	1	2	3	4	5	daily	weekly	monthly	occasionally	never	daily	weekly	monthly	occasionally	never
Pain	1	2	3	4	5	daily	weekly	monthly	occasionally	never	daily	weekly	monthly	occasionally	never
Range of motion of arm/leg	1	2	3	4	5	daily	weekly	monthly	occasionally	never	daily	weekly	monthly	occasionally	never
Patient's ability to self-exercise/self-practice	1	2	3	4	5	daily	weekly	monthly	occasionally	never	daily	weekly	monthly	occasionally	never
Other important health measures															
Other (please specify) ___________	1	2	3	4	5	daily	weekly	monthly	occasionally	never	daily	weekly	monthly	occasionally	never
Other (please specify) ___________	1	2	3	4	5	daily	weekly	monthly	occasionally	never	daily	weekly	monthly	occasionally	never

Part 2. Use of mobile apps in practices and preferred functions and health measurements for the management of people with stroke and TIA

9. As part of your usual practice/research, have you recommended **mobile apps** for managing health conditions and wellbeing, or carried out trials of apps?

   ☐ Yes

   ☐ No

   If yes, what purposes and/or health conditions were these apps used for? (check all that are relevant)

   ☐ Behaviour management (e.g., smoking cessation)

   ☐ Managing specific medical conditions (please specify)______

   ☐ Medication management

   ☐ Mental health support (e.g., anxiety, depression, mindfulness)

   ☐ Physical activity monitoring (e.g., step counting, workout management)

   ☐ Other (please specify)________

   If yes, where did you learn about the mobile apps that you recommended/prescribed or have trialled? (check all that apply)

   ☐ Found through my own research

   ☐ Recommended by other health care providers

   ☐ Suggested by my hospital/institute

   ☐ Supported by research trial

   ☐ Other (please specify)_______

10. If we design a multicomponent support program using digital health technology that includes a **mobile app** to facilitate guiding the ongoing care and support of people with stroke/TIA. Please rate the following functions you feel should be included and then rank the top five most important functions from 1 (most important) to 5.

**Functions**	**How important**					**Rank the FIVE most important functions.**
	**Not important at all (1)**	**Not very important (2)**	**Neutral (3)**	**Somewhat important (4)**	**Very important (5)**	
Alerting clinicians about potential deterioration in a patient's stroke/TIA risk/condition	1	2	3	4	5	
Alerting patients about potential deterioration in their stroke/TIA risk before they would normally notice	1	2	3	4	5	
Collection and monitoring lifestyle measure (e.g., physical exercises, diet, alcohol, smoke)	1	2	3	4	5	
Collection and monitoring of medical measures (e.g., pain, blood pressure)	1	2	3	4	5	
Educational information about stroke/TIA treatment and prevention in general	1	2	3	4	5	
Receiving persuasive messages to encourage compliance to rehabilitation programs	1	2	3	4	5	
Receiving virtual medals (awards) for compliance achievement	1	2	3	4	5	
Reminders (e.g., appointment)	1	2	3	4	5	
Reminders about the warning signs of stroke/TIA and what to do	1	2	3	4	5	
Supporting clinician-patient communication (via messages **from** patients)	1	2	3	4	5	
Supporting clinician-patient communication (via messages **to** patients)	1	2	3	4	5	

Supporting clinicians to review patients' compliance with their rehabilitation programs	1	2	3	4	5	
Supporting information sharing between clinicians (e.g., via messages)	1	2	3	4	5	
Tools to manage adaption to new lifestyle, (e.g., physical exercises, diet, alcohol, smoke)	1	2	3	4	5	
Tools to manage medication	1	2	3	4	5	
Tools to manage mental health	1	2	3	4	5	
Other, please specify_____	1	2	3	4	5	

11. If a digital health support program were available to assist secondary prevention and management of people with stroke/TIA, please rate the following types of measures would you want to be included from **mobile apps**.

**Attributes**	**Not important at all (1)**	**Not very important (2)**	**Neutral (3)**	**Somewhat important (4)**	**Very important (5)**
Anthropometrics such as weight or BMI	1	2	3	4	5
Lifestyle such as alcohol consumption, tobacco use, sedentary lifestyle, physical activity, diet and nutrition	1	2	3	4	5
Mental health, including stress, anxiety, depression management	1	2	3	4	5
Physiological monitoring such as sleep, heart rate, blood pressure	1	2	3	4	5
Mobility including pain and range of movement	1	2	3	4	5
Other (please specify) _______	1	2	3	4	5

Part 3. Wearable and health monitoring devices and preferred health measurements for monitoring secondary prevention and support for patients with stroke/TIA

12. **Wearable and health monitoring** devices are increasingly being used to manage acute and chronic health conditions, both through prescription from a health provider and through the patient's own initiative. Please rank the top three most important measures (from 1: most important to 3) you feel would provide value and utility in your management and secondary prevention of people with stroke/TIA if they are monitored/collected regularly by individuals in their own home.

**Measures**	**Rank the top three most important measures**
Blood glucose	
Blood oxygen (i.e. pulse oximeter)	
Blood pressure	
ECG/heart rate/rhythm (e.g., Polar Band)	
Exercise (i.e. steps, stairs, amount of time doing moderately vigorous and vigorous physical exercise)	
Falls (i.e. fall detection)	
Pain	
Range of motion (e.g., arm, leg)	
Sleep (hours of duration, quality)	
Temperature (i.e. thermometer)	
Weight (i.e. scale)	
Other (please specify)_______	

Part 4. Perception of using mobile health technologies to support stroke/TIA care

13. If a digital support program were available that collected individual health data via an app and summarised their digital health information from their wearables and other health monitoring devices, **how often and how much time** (per person) would you be able to spend reviewing the data collected in your clinical practice and follow-up of individuals with stroke/TIA? Please indicate the response that best applies.

- ☐ Never, I'm too busy
- ☐ Only during patients' consultations
- ☐ Daily for _______minutes
- ☐ Weekly for_______minutes
- ☐ Occasionally for_______minutes
- ☐ Other (please specify)_______

14. How would you like to access the data collected from apps and wearables and other health monitoring devices for your clients?

Accessing monitoring health data	Tick all that are applicable
Via web-based portal	☐
Via mobile app installed on smartphone or tablet	☐
Both web-based portal and mobile app	☐
Via the patient's app, during their consultation	☐
I'm not interested in looking at the data	☐

Other (please specify) ___________

15. When thinking about a new digital support program for use by people with stroke or TIA, how **important** it is for these items to be considered?

Items	How important				
	**Not important at all (1)**	**Not very important (2)**	**Neutral (3)**	**Somewhat important (4)**	**Very important (5)**
Accuracy of the data collected	1	2	3	4	5
Availability of technical support	1	2	3	4	5
Cost (affordable)	1	2	3	4	5
Easy to integrate with other devices and technologies	1	2	3	4	5
Easy to use	1	2	3	4	5
Privacy of the data collected	1	2	3	4	5
Sharing of the data with clinicians	1	2	3	4	5
Sharing of the data with patients' family member/carer	1	2	3	4	5
Tailored to address patients' care needs	1	2	3	4	5

16. If a **new support program that used digital technologies were available now** to support the care you provide to individuals with stroke/TIA, please rate how much you agree with each of the following statements?

**Items**	**Answers**				
	**Strongly disagree**	**Disagree**	**Neutral**	**Agree**	**Strongly agree**
Perceived usefulness					
Using a digital program could improve recovery after stroke or TIA	1	2	3	4	5
Using a digital program could make it easier to manage the health and wellbeing of people with stroke/TIA	1	2	3	4	5
Using a digital program could help in communicating with my patients/clients	1	2	3	4	5
Using a digital program could be useful to monitor the health and wellbeing of individuals with stroke/TIA	1	2	3	4	5
Using a digital program would be useful in supporting individuals adapt to a new lifestyle for secondary prevention of stroke/TIA	1	2	3	4	5
Perceived ease of use					
Learning to use a digital program would be easy for me	1	2	3	4	5
I would be able to use a digital program without much effort	1	2	3	4	5
It would be easy for me to become skilful in using a digital program	1	2	3	4	5

Social influence					
My colleagues would expect me to use a digital program	1	2	3	4	5
My direct manager(s) would be supportive of my use of a digital program	1	2	3	4	5
Other clinicians who influence my clinical behaviour would think that I should use a digital program	1	2	3	4	5
My clients/patients with stroke/TIA would support my use of a digital program	1	2	3	4	5
Intention to use					
I would intend to use the digital program in my care of people with stroke/TIA	1	2	3	4	5
I would intend to use the digital program as often as needed	1	2	3	4	5
I would intend to use the digital program daily	1	2	3	4	5
I would intend to discuss with the individual, the data collected in the digital program when I have face-to-face contact with them	1	2	3	4	5
Personal innovativeness					
If I hear about a new technology for health care, I usually look for ways to experiment with it	1	2	3	4	5
Among my peers, I am usually the first to try out new technologies for health care	1	2	3	4	5

**Self-efficacy**					
Using a digital program would be somewhat intimidating for me	1	2	3	4	5
I would be hesitant to use a digital program for fear of making mistakes that I cannot correct	1	2	3	4	5
I *would* feel apprehensive about using a digital program	1	2	3	4	5
**Compatibility**					
A digital health program would fit in well with the way I like to work	1	2	3	4	5
A digital health program would be compatible with most aspects of my work	1	2	3	4	5

17. Do you have any comments or suggestions for the design features for a digital health support program for people living with stroke and TIA?

____

18. Would you be willing to be contacted to be further involved in the study such as workshops to design and test the features of the digital health support program, for people living with stroke or TIA?

☐ Yes, can you please provide your best contact below ☐ No

Name:
Contact number:
Email:

Thank you for your time in completing this survey.
