# Supplementary material for: Multicomponent Support Program for Secondary Prevention of Stroke Using Digital Health Technology: Co-Design Study With People Living With Stroke or Transient Ischemic Attack
Source: J Med Internet Res. 2024 Aug 22;26:e54604. doi: 10.2196/54604 (PMC11377903; doi:10.2196/54604)
Supplement: Multimedia Appendix 3 [file jmir_v26i1e54604_app3.pdf]

	All	Medical Doctor	Nurse	OT	Physio	Other
	n=49	n=12	n=11	N=7	n=11	n=8
**At least 1 measure per participant**	**46 (94%)**	**12 (100%)**	**9 (82%)**	**7 (100%)**	**11 (100%)**	**7 (88%)**
Waist circumference: n (%)	33 (67)	9 (75)	8 (73)	6 (86)	6 (55)	4 (50)
Dietary intake: n (%)	26 (53)	7 (58)	3 (27)	6 (86)	6 (55)	4 (50)
Sedentary lifestyle: n (%)	26 (53)	5 (42)	3 (27)	7 (100)	7 (64)	4 (50)
Body mass index: n (%)	25 (51)	8 (67)	4 (36)	4 (57)	6 (55)	3 (38)
Intensive physical activity: n (%)	25 (51)	7 (58)	4 (36)	4 (57)	7 (64)	3 (38)
Sleep hours: n (%)	24 (49)	6 (50)	4 (36)	6 (86)	6 (55)	2 (25)
Physical activity: n (%)	22 (45)	5 (42)	2 (18)	4 (57)	7 (64)	4 (50)
Continuous heart rate: n (%)	22 (45)	6 (50)	2 (18)	6 (86)	6 (55)	2 (25)
Weight: n (%)	21 (43)	5 (42)	4 (36)	4 (57)	4 (36)	4 (50)
Ability to self-exercise: n (%)	20 (41)	4 (33)	2 (18)	4 (57)	5 (45)	5 (63)
ECG: n (%)	18 (37)	1 (8)	4 (36)	3 (43)	7 (64)	3 (38)
Blood oxygen: n (%)	16 (33)	4 (33)	1 (9)	5 (71)	3 (27)	3 (38)
Number of falls: n (%)	16 (33)	4 (33)	3 (27)	4 (57)	4 (36)	1 (13)
Resting heart rate: n (%)	15 (31)	5 (42)	1 (9)	5 (71)	2 (18)	2 (25)
Blood glucose: n (%)	15 (31)	2 (17)	1 (9)	5 (71)	4 (36)	3 (38)
Pain: n (%)	15 (31)	6 (50)	2 (18)	4 (57)	1 (9)	2 (25)
Alcohol consumption: n (%)	13 (27)	1 (8)	2 (18)	5 (71)	2 (18)	3 (38)
Tobacco use: n (%)	13 (27)	1 (8)	1 (9)	4 (57)	3 (27)	4 (50)
Blood pressure: n (%)	11 (22)	3 (25)	1 (9)	4 (57)	2 (18)	1 (13)
Range of motion: n (%)	9 (18)	2 (17)	1 (9)	3 (43)	1 (9)	2 (25)
