# Supplementary figures and images for: Multicomponent Support Program for Secondary Prevention of Stroke Using Digital Health Technology: Co-Design Study With People Living With Stroke or Transient Ischemic Attack
Source: J Med Internet Res. 2024 Aug 22;26:e54604. doi: 10.2196/54604 (PMC11377903; doi:10.2196/54604)

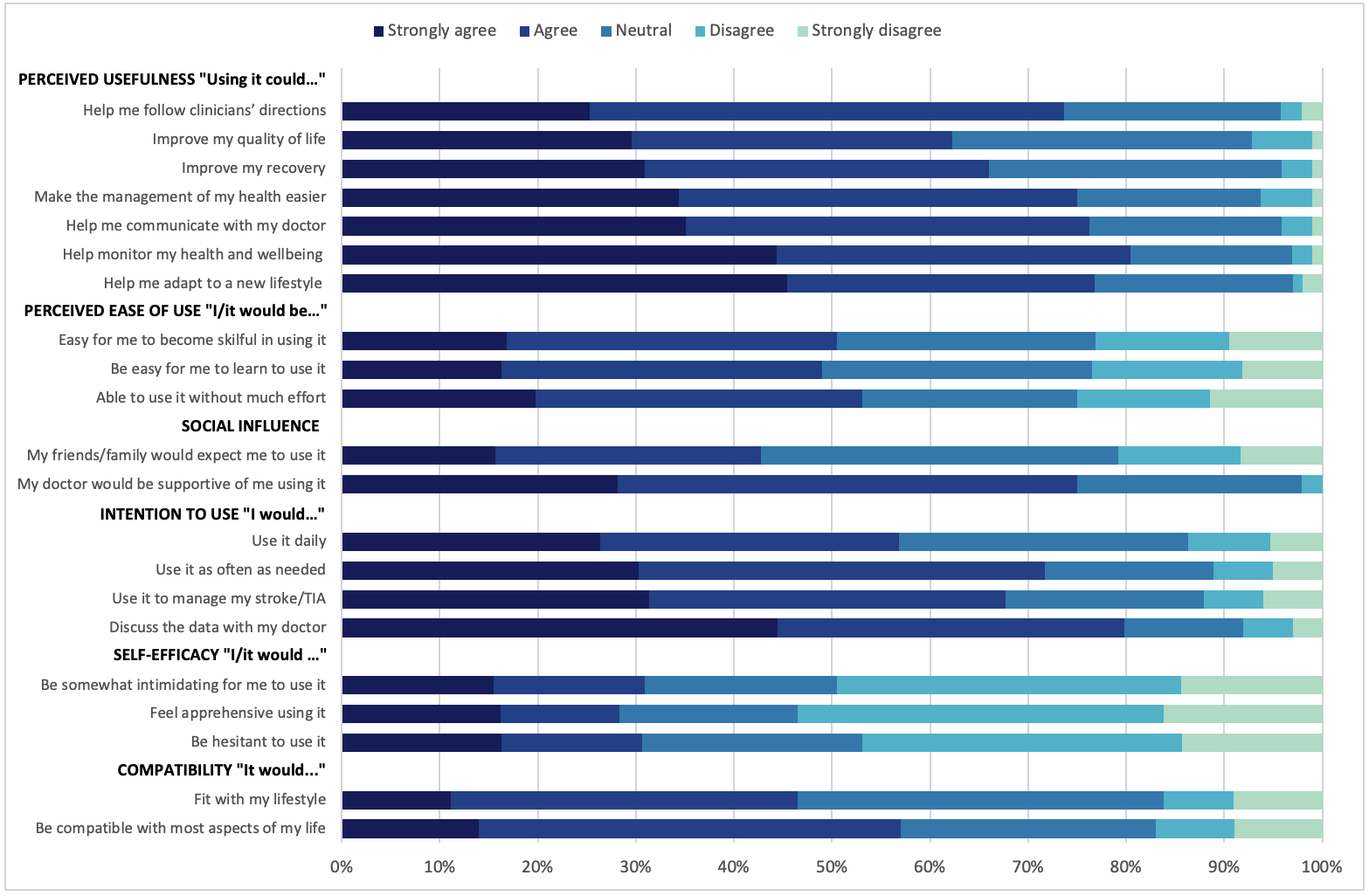

Supplement: Multimedia Appendix 4 [file jmir_v26i1e54604_app4.png]

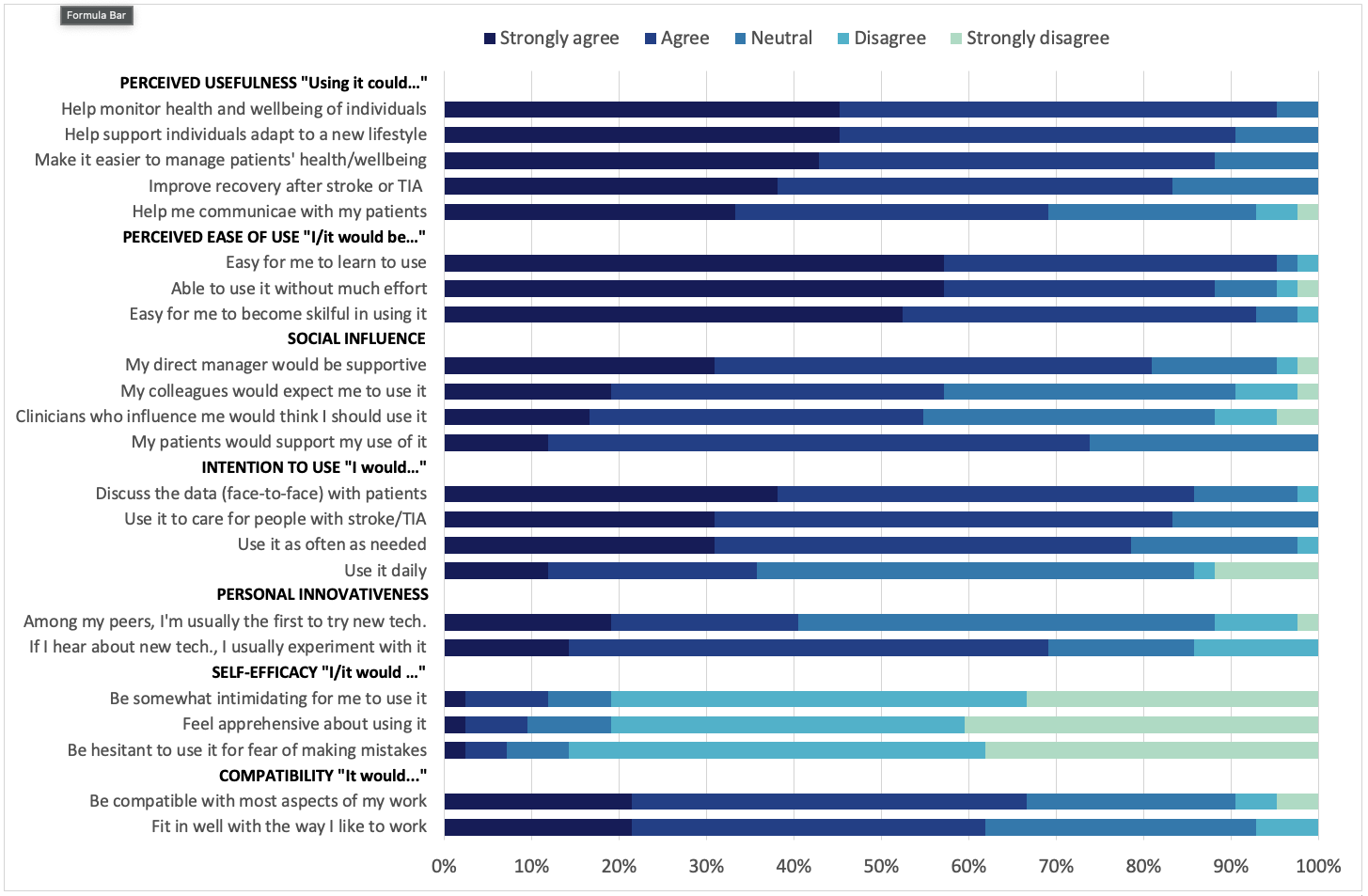

Supplement: Multimedia Appendix 5 [file jmir_v26i1e54604_app5.png]
